# Identifying AWaRe indicators for appropriate antibiotic use: a narrative review

**DOI:** 10.1093/jac/dkae370

**Published:** 2024-10-18

**Authors:** Elisa Funiciello, Giulia Lorenzetti, Aislinn Cook, Jan Goelen, Catrin E Moore, Stephen M Campbell, Brian Godman, Deborah Tong, Benedikt Huttner, Pem Chuki, Michael Sharland

**Affiliations:** Centre for Neonatal and Paediatric Infection, St. George’s University of London, London SW17 0RE, UK; Centre for Neonatal and Paediatric Infection, St. George’s University of London, London SW17 0RE, UK; Centre for Neonatal and Paediatric Infection, St. George’s University of London, London SW17 0RE, UK; Health Economics Research Centre, Nuffield, Department of Population Health, University of Oxford, Oxford OX1 2JD, UK; Centre for Neonatal and Paediatric Infection, St. George’s University of London, London SW17 0RE, UK; Centre for Neonatal and Paediatric Infection, St. George’s University of London, London SW17 0RE, UK; School of Health Sciences, University of Manchester, Manchester M13 9PL, UK; School of Pharmacy, Sefako Makgatho Health Sciences University, Ga-Rankuwa, Pretoria 0208, South Africa; School of Pharmacy, Sefako Makgatho Health Sciences University, Ga-Rankuwa, Pretoria 0208, South Africa; Strathclyde Institute of Pharmacy and Biomedical Sciences, Strathclyde University, Glasgow G4 0RE, UK; Department of Surveillance, Prevention and Control, Division of Antimicrobial Resistance, World Health Organization, Avenue Appia 20, 1211 Geneva, Switzerland; Department of Health Products Policy and Standards, World Health Organization, Avenue Appia 20, 1211 Geneva, Switzerland; Antimicrobial Stewardship Unit, Jigme Dorji Wangchuck National Referral Hospital, Thimphu, Bhutan; Centre for Neonatal and Paediatric Infection, St. George’s University of London, London SW17 0RE, UK

## Abstract

**Introduction:**

Quality indicators (QIs) are widely used tools for antibiotic stewardship programmes. The Access, Watch, Reserve (AWaRe) system has been developed by the WHO to classify antibiotics based on their spectrum of activity and potential selection of antibiotic resistance. This review aimed to identify existing indicators for optimal antibiotic use to inform the development of future AWaRe QIs.

**Methods:**

A literature search was performed in PubMed. We included articles describing QIs for hospital and primary healthcare antibiotic use. We extracted information about (i) the type of infection; (ii) setting; (iii) target for quality assessment; and (iv) methodology used for the development. We then identified the indicators that reflected the guidance provided in the AWaRe system.

**Results:**

A total of 773 indicators for antibiotic use were identified. The management of health services and/or workers, the consumption of antibiotics, and antibiotic prescribing/dispensing were the principal targets for quality assessment. There was a similar distribution of indicators across primary and secondary care. For infection-specific indicators, about 50% focused on respiratory tract infections. Only a few QIs included information on review treatment or microbiological investigations. Although only 8 (1%) indicators directly cited the AWaRe system in the wording of the indicators, 445 (57.6%) indicators reflected the guidance provided in the AWaRe book.

**Conclusions:**

A high number of indicators for appropriate antibiotic use have been developed. However, few are currently based directly on the WHO AWaRe system. There is a clear need to develop globally applicable AWaRe based indicators that can be integrated into antibiotic stewardship programmes.

## Background

Antimicrobial resistance (AMR) poses a significant global threat to public health.^1^ The inappropriate use of antibiotics both in terms of choice and volume is an important driver behind this health emergency and reducing inappropriate use is important in tackling AMR.^2^ Consequently, monitoring consumption and appropriateness of antibiotic use is a priority as highlighted in the Global Action Plan (GAP) on AMR (Table 1).^3,4^ Towards this, the WHO established the Access-Watch-Reserve (AWaRe) system in 2017 as an antimicrobial stewardship tool, in which antibiotics are classified into four groups (Access, Watch, Reserve and Not Recommended) based on their spectrum of activity and potential selection for resistance.^5^ The WHO AWaRe antibiotic book (AWaRe book) was published in 2022,^6^ guiding the diagnosis and treatment of the 34 most common infections in primary health care and hospitals, in alignment with the recommendations for antibiotics included in the WHO Model List of Essential Medicines and Essential Medicines for Children.^7,8^

**Table 1.** Indicators from the monitoring and evaluation of the GAP on AMR relevant to humans^4^

OUTCOME 4: Optimized use of antimicrobials in human and animal health		
Measurement	Indicator name	Source of data at the global level
4.1 Use of antimicrobials in humans	Total human consumption of antibiotics for systemic use (Anatomical Therapeutic Chemical classification code J01) in Defined Daily Doses per 1000 population (or inhabitants) per day Proportion of Access antibiotics for systemic use, relative to total antibiotic consumption in Defined Daily Doses Relative proportion of AWaRe antibiotics for paediatric formulations Percentage of adult and paediatric hospital patients receiving an antibiotic according to AWaRe categories	Global antimicrobial resistance and use surveillance system (GLASS) Cross-sectional point prevalence survey
4.2 Access to antibiotics	Percentage of health facilities that have a core set of relevant antibiotics available and affordable on a sustainable basis	Sustainable Development Goal indicator 3.b.3, with Access antibiotics disaggregated
4.3 Appropriate use of antimicrobials	Percentage of inpatient surgical procedures with appropriate timing and duration of surgical antibiotic prophylaxis	Point prevalence surveys
4.7 Optimized AMU and regulation	Legislation or regulation that requires antimicrobials for human use to be dispensed only with a prescription from an authorized health worker	Tracking AMR country self-assessment survey

Quality indicators (QIs) have been developed for different healthcare areas, including antibiotic prescribing,^9^ and are able to reflect the degree to which an antibiotic is clinically indicated and appropriate. Quantity metrics are quantifiable measures used to assess the performance, effectiveness, and overall quality of a process, service, or system such as reflecting the volume of antibiotic use but they focus primarily on quantity rather than the direct quality of care or single measurable elements of care.^10^ QIs focus on discrete single issues or processes as measurable elements of care that provide an indication of the *quality of care* as a standardized, evidence-based measure of health care quality using routinely available data to measure and track clinical performance and outcomes. QIs generally have an associated target or achieved ‘standard’ giving an indication of good or poor quality, which can be used to show and track differences and changes in quality.^11^ It is important that indicators adhere to essential measurement attributes to ensure clearly defined, objective, evidence-based, measurable, reliable, valid and feasible quality assessment^11–15^ that mean that they are likely to be valid and feasible across varying localities and countries.^16^ QIs are crucial components of antimicrobial stewardship programmes (ASPs). These quality assessment tools are essential for improving quality of care and for indicating the extent to which a healthcare system meets the needs of patients, they enhance treatment outcomes, while reducing the selection of antibiotic resistance and limiting the costs of healthcare and treatment regimens.^1,3,4^

The recent publication of the AWaRe book provides an opportunity for developing a common set of agreed AWaRe QIs across sectors and countries in combination with indicators published in existing literature. To refine the scope of potential future AWaRe QIs, we performed a narrative review of existing indicators.

This review aimed to identify published QIs evaluating the appropriateness of antibiotic use in hospital and primary healthcare settings. As a secondary objective, we evaluated the proportion of current indicators that were based directly on or reflected the guidance of the AWaRe system.

## Methods

### Search strategy

We searched the MEDLINE database using PubMed for articles describing QIs for hospital and primary health care antibiotic use published from 1 January 1996 up to 1 March 2023. The search strategy is shown in Figure S1 (available as Supplementary data at *JAC* Online). The reference lists of all included articles were screened manually for additional relevant papers. A manual search of the grey literature was also conducted together with websites (in English) from 26 national and international infectious disease societies and public health organizations (Table S1). Two reviewers (G.L. and E.F.) screened these websites using ‘indicator or metric’ with or without ‘antibiotics or antimicrobials’ as search terms.

### Screening process and data collection

Articles published in English focusing on systemic (oral or IV) antibiotic use describing QIs were included. We included all populations; adults and/or children attending community and/or hospital healthcare facilities in high-, low- and middle-income countries (HIC and LMICs). Articles on the use of antiviral, antifungal, antiparasitic or antituberculosis drugs were excluded.

Titles, abstracts, and articles were reviewed by a single investigator (G.L.). Two investigators (G.L. and E.F.) extracted data using a standardized form and eliminated duplicates and indicators not focused on antibiotics. Data on relevant indicators were collected and classified as ‘Clinical’ (e.g. choice of antibiotic or performance of diagnostic tests such as ‘Outpatients with an acute tonsillitis/pharyngitis and positive Group A streptococcal diagnostic test should be treated with antibiotics’), ‘Organizational’ (e.g. recording of data, premises/facilities management such as ‘Prophylactic antibiotics should be added to a pre-operative checklist’), and ‘Workforce’ (i.e. focused on health workers, e.g. ‘Each member of the Outpatient Parenteral Antimicrobial Therapy team is responsible for personal continuing professional development relating to best clinical practice’) indicators. This classification was carried out by a team of seven members (M.S., C.e.M., S.M.C., A.C., E.F., G.L. and J.G.) with additional expertise in infectious diseases epidemiology, healthcare, public health and antimicrobial stewardship. The final set of indicators was divided into five subgroups based on setting: ‘Hospital facility’, ‘Primary Health Care’, ‘Both Hospital and Primary Health Care’, ‘Outpatient parenteral antibiotic therapy’ and ‘General indicators’. General indicators were defined as those not specific to any particular disease and/or setting [e.g. ‘Antibiotics should not be prescribed for (most) viral infections or self-limiting bacterial infections’^17^]. To describe and compare the identified indicators, information on the type of infection, and the target for quality assessment were analysed. Among the latter we identified five categories: (i) antibiotic prescribing/dispensing (i.e. indicators focusing on the decision to prescribe antibiotics and/or the choice, dose, review, and duration of antibiotic therapy), (ii) consumption of antibiotics/prescription rate, (iii) diagnostic process (i.e. indicators focusing on laboratory, microbiological or radiological assessment), (iv) management (i.e. indicators focusing on the organisation of health services, health workers, and staff tasks/workforce), (v) outcomes (e.g. ‘Pneumonia mortality rate’).

Indicators that were specifically based on the AWaRe system (i.e. indicators in which the AWaRe classification or the AWaRe book were cited) and indicators that reflected the contents and treatment recommendations of the AWaRe book were included. Indicators were defined as ‘non-AWaRe indicators’ if they were focused on topics not explicitly taken into account by the AWaRe system such as national/regional/local policies (e.g. ‘The local guidelines should correspond to the national guideline but should be adapted based on local resistance patterns’), other settings (e.g. outpatient parenteral antibiotic therapy), specific clinical diseases (e.g. otitis externa), laboratory tests (e.g. therapeutic drug monitoring) and/or specific therapies (e.g. topical preparations).

## Results

### Search results and study characteristics

**Figure 1.**
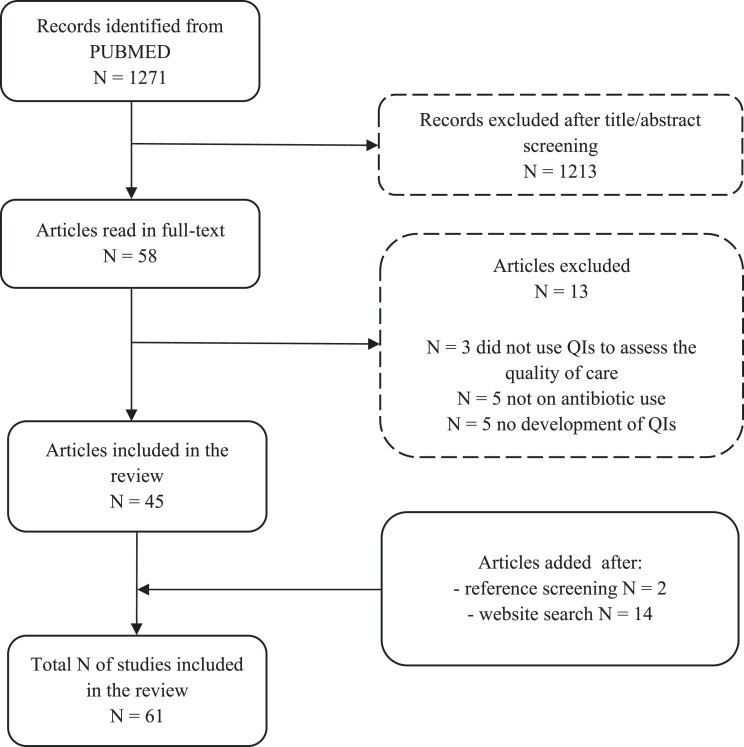
Flow diagram summary of the paper selection process.

The literature search of MEDLINE identified 1271 studies. After Title/Abstract screening, 58 potentially relevant studies were selected for full-text screening. Of these, 13 were excluded as no indicators assessed the quality of care (*n* = 3) or concerned antibiotic use (*n* = 5) or the development of indicators (*n* = 5). We added 2 studies and 14 websites after screening the reference list of all included articles and the principal infectious disease societies and public health organisations’ websites (Table S1). The selection process resulted in a total of 61 studies and guidelines fulfilling the criteria for synthesis in this review (Figure 1).^4,13,17–75^ Table 2 provides an overview of all papers included in this review.

**Table 2.** Overview of studies reporting QIs for appropriate antibiotic use

First author, year	Consensus methods	Evidence base	Stakeholder involvement	Focus/population (when specified)	No. of indicators	Application (when specified)
Hospital care						
Afriyie, 2019^18^	NA	Indicators based on the GPPSS and other studies	NA	NA	5	International
Berenholtz, 2007^19^	Modified Nominal Group Technique	Literature review (MEDLINE via PubMed)	National (Dutch) multidisciplinary panel from multiple hospitals	Sepsis care in intensive care unit (adults-age > 16 years)	6	NA
Bramesfeld, 2015^20^	Modified RAND/UCLA appropriateness method	Systematic literature review (EMBASE) + additional international databases	National (Germany) multidisciplinary panel of 13 experts + 2 representatives of federal patient organisations	Prevention and management of central venous catheter-related bloodstream infections (adults + children)	32	National (Germany)
Buyle, 2013^21^	Non-Delphi method (three-step procedure—described)	Literature review	Multidisciplinary panel of 13 international experts from 4 European countries	Antimicrobial stewardship programmes evaluation	58 (minimal set of 10 key structure indicators)	Europe
Coll, 2012^22^	Non-Delphi method (multidisciplinary team agreement)	Literature review, national guidelines, local policy	Multidisciplinary team in one UK hospital	Inpatient setting	30	National (UK)
Farida, 2015^23^	Two-round Delphi procedure	National guidelines + indicators by Schouten et al.^24^	National (Indonesia) multidisciplinary panel of 18 experts from multiple hospitals	Community-acquired pneumonia care in hospitalized patients	6	Middle-income developing countries (Indonesia)
Schouten, 2005^24^	Four-step RAND-modified Delphi procedure	Systematic literature review (PubMed) + national and international guidelines	Multidisciplinary panel of 11 experts	CAP and acute exacerbation of chronic bronchitis or chronic obstructive pulmonary disease care in hospitalized adults	15	NA
Harvey, 2023^25^	Four-step Delphi procedure	Literature review, individual hospital policies, expert advice	National (UK) multidisciplinary panel of experts	Antimicrobial IV-to-oral switch (IVOS) criteria in hospital setting (adults)	12	National (UK)
Hermanides, 2008^26^	Three-step RAND-modified Delphi procedure	National guideline for treatment of complicated UTI in adults	National (Dutch) multidisciplinary panel of 13 experts from multiple hospitals	Complicated urinary tract infection care in the hospital setting (adults)	13	NA
Kallen, 2018^27^	Four-round modified-RAND Delphi procedure	Systematic literature review (MEDLINE) + international guideline search	National (Dutch) multidisciplinary panel of 15 experts from multiple hospitals	Intensive care unit (adults)	5	NA
Kim, 2021^28^	Four-step RAND-modified Delphi procedure	Systematic literature review (PubMed, EMBASE, Cochrane)	National (Korea) multidisciplinary panel of 25 experts from multiple hospitals	Inpatients, Prophylaxis (adults + children)	8	National (Korea)
Li, 2017^29^	Three-round Delphi procedure	Literature search (PubMed, EMBASE, Cochrane), China Biology Medicine disc (CBM), National guidelines	National (China) multidisciplinary panel of 22 experts from multiple hospitals	CAP care in hospitals and clinics (Children)	21	National (China)
Monnier, 2018 (DRIVE-AB)^30^	Four-step RAND-modified Delphi procedure	Systematic literature review (MEDLINE) + web site search	Multidisciplinary panel of 51 international experts from 15 countries	Inpatient setting (including ICU), Surgical prophylaxis (adults + children)	51	International
Morris, 2012^31^	RAND-modified Delphi procedure	Literature review	Multidisciplinary panel of 10 international experts from Canada and USA	Antimicrobial stewardship programmes evaluation	5	International
Oduyebo, 2018^32^	NA	Indicators based on other studies	NA	Inpatient setting (including ICU), Surgical Prophylaxis (Adults)	3	International
Okoth, 2019^33^	NA	Indicators based on other studies	NA	Indicators based on other studies	6	International
Pollack, 2016^34^	RAND/UCLA-modified Delphi procedure	Literature review, list of indicators by Davey et al., guidelines in the European Union and USA	Multidisciplinary panel of 20 international experts	Antimicrobial stewardship programmes evaluation	33	USA and EU
Pulcini, 2008^35^	Non-Delphi method (team agreement)	Literature review focused on guidelines	Panel of 3 infectious diseases experts	Assessment of inpatient empirical antibiotic prescriptions	5	NA
Pulcini, 2019^36^	Three-step modified Delphi procedure	Literature review (MEDLINE) + web site search	Multidisciplinary panel of 15 international experts (13 countries in 6 continents)	Antimicrobial stewardship programmes evaluation	7 core elements + 29 related checklist items	International
Schoffelen, 2021^37^	RAND-modified Delphi procedure	DRIVE-AB outpatients and inpatients QIs	Multidisciplinary panel of 13 international experts from 7 countries	Emergency Department (adults)	22	International
Science, 2016^38^	Modified Delphi procedure	Literature review	National (Canada) multidisciplinary panel of 38 experts from multiple paediatric hospitals	Paediatric Antimicrobial stewardship programmes evaluation in Canada (Children)	4	International
Stanić Benić, 2018 (DRIVE-AB)^39^	Four-step RAND-modified Delphi procedure	Systematic literature review (MEDLINE) + web site search	Multidisciplinary panel of 23 international stakeholders	Inpatient setting	12	International
Skosana, 2021^40^	NA	Indicators based on other studies	NA	Inpatient setting (including ICU), Surgical prophylaxis (adults)	3	International
ten Oever, 2019^41^	RAND-modified Delphi procedure	Systematic literature review (MEDLINE, EMBASE)	Multidisciplinary panel of 30 international experts	Management of *Staphylococcus aureus* bacteraemia in hospitalized patients (adults)	15	NA
Thern, 2014^42^	Three-step RAND/UCLA-modified Delphi procedure	Extensive literature review, national guidelines	National (Germany) multidisciplinary panel of experts from multiple hospitals	Hospital antimicrobial stewardship and infection management in the inpatient setting	42	National (Germany)
van den Bosch, 2014^43^	Five-step RAND-modified Delphi procedure	National guideline for antimicrobial use in hospitalized patients with sepsis	National (Dutch) multidisciplinary panel of 14 experts from multiple hospitals	Sepsis care in patients hospitalized in general medical ward or ICU (Adults)	5	National (Netherlands)
van den Bosch, 2015^44^	Four-step RAND-modified Delphi procedure	Literature review (PubMed, EMBASE)	Multidisciplinary panel of 17 international experts from 6 European countries	Inpatient setting, excluding ICU (Adults)	11	International
Vera, 2014^45^	NA	Literature review and guidelines by Spanish working group of Infectious Diseases	NA	Critically ill patients admitted to ICU	10	NA
Ambulatory care						
Adriaenssens, 2011 (ESAC-Net)^46^	RAND/UCLA Appropriateness Method, 2 rounds of scoring	Workshop of experts from different research groups and projects, guidelines	Multidisciplinary panel of international experts from 24 European countries and Israel	Outpatient setting (adults + children)	21	Europe
Bateman, 1996^47^	Non-Delphi method (team agreement)	National guidelines	Panel of 8 UK general practitioners	General practice	1	National (UK)
Berrevoets, 2020^48^	Four-step RAND-modified Delphi procedure	Systematic literature review (MEDLINE via PubMed, EMBASE, Cochrane)	Multidisciplinary panel of 19 international experts	OPAT (adults)	33 (12 prioritized)	International
Campbell, 2000^49^	Two-round Delphi procedure	Previous studies, prescribing analysis and cost (PACT) data	Multidisciplinary panel (health authority medical and pharmaceutical advisers)	General practice	4	National (UK)
Coenen, 2007 (ESAC-Net)^50^	RAND/UCLA appropriateness method, two rounds of scoring	Workshop of experts, ESAC data on antibiotic consumption	Multidisciplinary panel of 22 international experts from 12 European countries	Outpatient setting (adults)	12	Europe
Cottrell, 2020 (rhinosinusitis)^51^	RAND/UCLA appropriateness method	Literature review, international guidelines	Multidisciplinary panel of 9 experts	Diagnosis and management of patients with acute bacterial rhinosinusitis (adults)	2	International
Cottrell, 2020 (tonsillitis)^52^	RAND/UCLA appropriateness method	Literature review, international guidelines	National (Canada) multidisciplinary panel of 11 experts	Diagnosis and management of Paediatric patients with tonsillitis (Children)	5	NA
de Bie, 2016^53^	Non-Delphi method (team agreement)	Expert consensus	NA	Outpatient setting (children)	2	Europe
Fernández Urrusuno, 2008^54^	Non-Delphi method (multidisciplinary team agreement)	National guidelines, local resistance patterns, expert consensus	Multidisciplinary panel of experts	General practice prescribing patterns of RTIs and UTIs	5	National (Spain)
Giesen, 2007^55^	Non-Delphi method (three-step procedure—described)	National clinical guidelines for general practice	Panel of 6 Dutch general practitioners	Out-of-hours general practice in the Netherlands (Adults + Children)	6	National (Netherlands)
Hansen, 2010^56^	Modified 2-round Delphi procedure	Literature review, workshop of experts, national guidelines	Multidisciplinary panel of 27 international experts from 13 countries	RTIs in general practice (Adults + Children)	41	International
Hussein, 2017^57^	RAND/UCLA appropriateness method	Systematic literature review (MEDLINE, EMBASE, Cochrane), clinical guidelines	National (Germany) multidisciplinary panel of 11 dental experts (dentists, oral and maxillofacial surgeons)	Systemic antibiotics in dentistry (adults + children)	15	National (Germany)
Korom 2017^58^	Non-Delphi method (multidisciplinary team agreement over three rounds of meetings)	Literature review, Kenyan Ministry of Health guidelines	National (Kenia) multidisciplinary panel of experts	Management of UTIs in ambulatory setting	1	National (Kenia)
Le Maréchal, 2018 (DRIVE-AB)^17^	RAND-modified Delphi procedure	Systematic literature review (MEDLINE via PubMed) + web site search	Multidisciplinary panel of 25 international experts from 14 countries	Outpatient setting including OPAT (adults + children)	32 (12 OPAT)	International (high-, middle-, low-income settings)
Pulcini, 2013^59^	NA	Literature review + international guidelines	National (France) multidisciplinary panel of 3 experts	General Practice (Adults—age ≥16 years)	6	National (France)
Saust, 2017^60^	RAND/UCLA appropriateness method	National and international guidelines for management of RTIs	National (Denmark) multidisciplinary panel of 9 experts	General Practice (adults + children)	50	National (Denmark)
Smith, 2018^61^	Expert elicitations (2) and anonymous online prescriber survey	Literature review, National guidelines	Multidisciplinary panel of 9 experts	General Practice (adults + children)	12	National (UK)
van Roosmalen, 2007^62^	Non-Delphi method (multidisciplinary team agreement after iterated consensus rating procedure—not described)	National clinical guidelines by the Dutch College of General practitioners	NA	General practice (adults + children)	7	National (Netherlands)
Versporten, 2018 (DRIVE-AB)^63^	RAND-modified Delphi procedure	Systematic literature review (MEDLINE) + web site search	Multidisciplinary panel of 23 international experts from 4 continents	Outpatient setting (adults)	6	International

NA, not available; GPPS, Global Point Prevalence Survey; UK, United Kingdom; UTIs, urinary tract infections; CAP, community-acquired pneumonia; ICU, intensive care unit; USA, United States of America; OPAT, outpatient parenteral antimicrobial therapy; RTIs, respiratory tract infections.

### Selection and analysis of indicators

A total of 1104 indicators for antibiotic prescribing were identified, from which 264 duplicates (23.9%) and 67 irrelevant indicators (6.1%) were excluded: 27 were concerned with elements unrelated to the use of antibiotics (e.g. ‘Use of hand disinfectants in ICU setting’), 26 with venous/urinary catheter placement and management, 9 with drugs other than antibiotics, 3 with laboratory and microbiological tests, and 2 were performance indicators.

**Figure 2.**
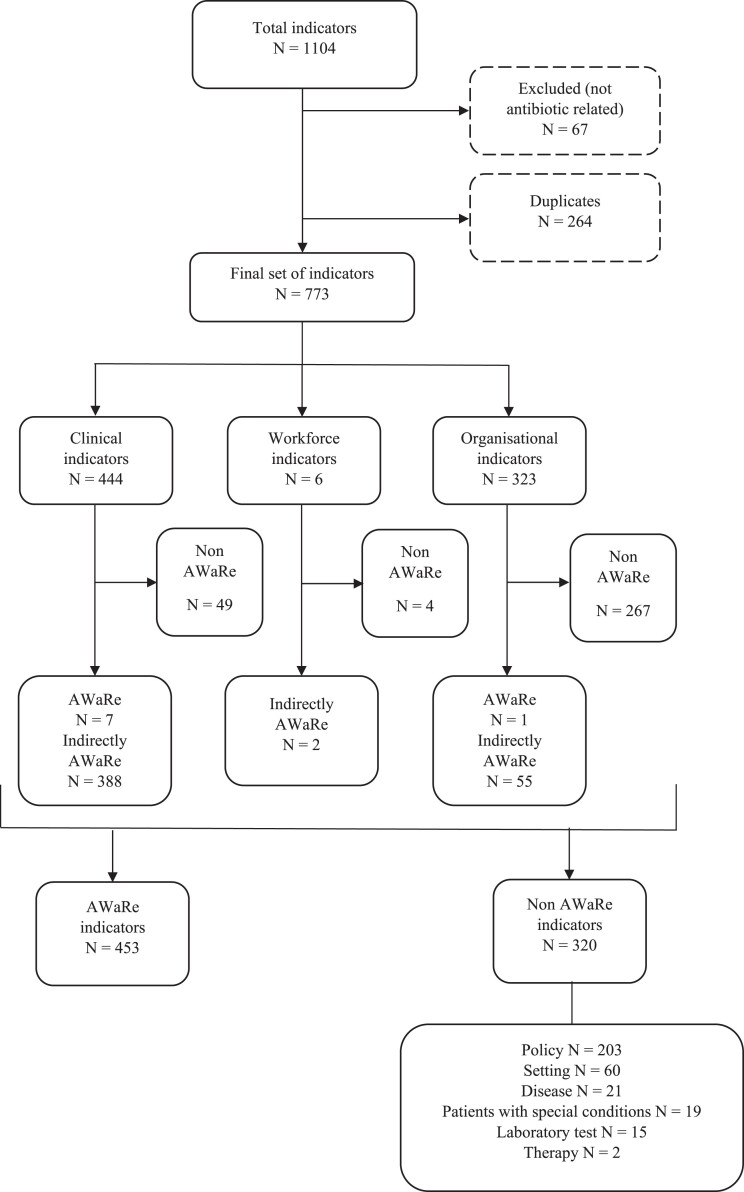
Flow diagram summary of the indicator selection process.

**Figure 3.**
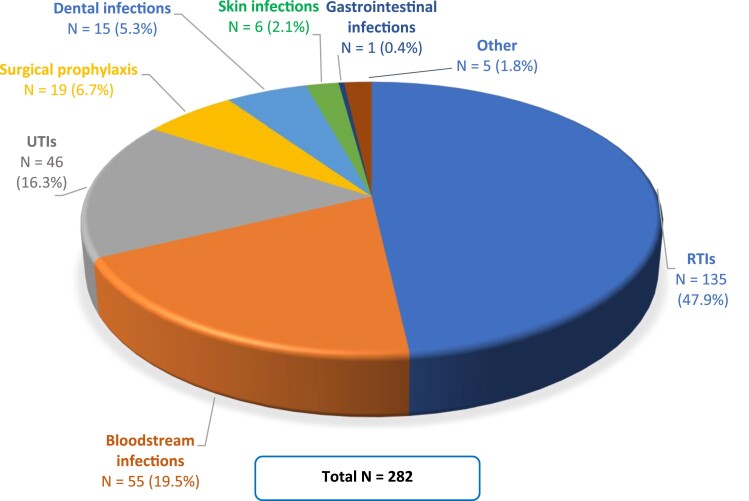
Indicators related to a specific type of infection. RTIs, respiratory tract infections; UTIs, urinary tract infections; Other, two or more different types of infection.

Figure 2 provides a flow diagram summary of the indicator selection process. Among the final set of indicators, 282/773 indicators (36.5%) referred to a specific type of infection, of which 135/282 (47.9%) were related to respiratory tract infections (RTIs), 55/282 (19.5%) to bloodstream infections, and 46/282 (16.3%) to urinary tract infections (Figure 3).

177/773 indicators (22.9%) were related to hospital facilities, 137/773 (17.7%) to primary health care, 44/773 (5.7%) to both hospital and primary health care, 60/773 (7.8%) to outpatient parenteral antibiotic therapy, and 355/773 (45.9%) were general indicators. Regarding the target for quality assessment, 206/773 indicators (26.6%) focused on antibiotic prescribing/dispensing (e.g. ‘Proportion of patients with no relevant comorbidities presenting with acute bronchitis that should be prescribed oral antibiotics’), 163/773 (21.1%) on the consumption of antibiotics/prescription rate (e.g. ‘Antimicrobial prescribing rates for men and non-pregnant women with asymptomatic bacteriuria’), 67/773 (8.7%) on the diagnostic process (e.g. ‘Number of patients with acute tonsillitis/pharyngitis treated with antibiotics with negative StrepA test’), 33/773 (4.3%) on the outcome (e.g. ‘Community Acquired Pneumonia Admission Rate’), and 304/773 (39.3%) on the management (e.g. ‘Indication for antimicrobial use (AMU) documented in the patient notes’) (Table 3).

**Table 3.** The final set of indicators related to the classification, setting and target for quality assessment

	AWaRe indicators (*N* = 8)	Indicators reflecting the AWaRe system/book (*N* = 445)	Indicators not related to the AWaRe system/book (*N* = 320)	Total (*N* = 773)
Classification *N* (%):				
Clinical	7 (87.5)	388 (87.2)	49 (15.3)	444 (57.4)
Organisational	1 (12.5)	55 (12.4)	267 (83.4)	323 (41.8)
Workforce	0 (0)	2 (0.4)	4 (1.3)	6 (0.8)
Setting *N* (%):				
Hospital facility	0 (0)	110 (24.8)	67 (20.9)	177 (22.9)
Primary health care	0 (0)	118 (26.5)	19 (5.9)	136 (17.6)
Both	0 (0)	34 (7.6)	10 (3.1)	45 (5.8)
OPAT	0 (0)	0 (0)	60 (18.7)	60 (7.8)
General	8 (100)	183 (41.1)	164 (51.4)	355 (45.9)
Target for quality assessment *N* (%):				
Antibiotic prescribing/dispensing	0 (0)	180 (40.4)	26 (8.1)	206 (26.6)
Consumption/prescription rate	7 (87.5)	156 (35.1)	0 (0)	163 (21.1)
Diagnostic process	0 (0)	63 (14.2)	4 (1.3)	67 (8.7)
Outcome	0 (0)	25 (5.6)	8 (2.5)	33 (4.3)
Management	1 (12.5)	21 (4.7)	282 (88.1)	304 (39.3)

OPAT, outpatient parenteral antibiotic therapy.

Among the antibiotic prescribing indicators (*n* = 206), 93 concerned the type of antibiotic, 54 the duration of therapy, 45 the timing of administration, 45 the route of administration, 26 therapy revision (i.e. reduction of the spectrum and/or switching from IV to oral therapy), 23 the decision to prescribe antibiotics and 5 the dose. 25/67 indicators (37.3%) focused on the diagnostic process related to microbiological investigations.

Only 8/773 indicators (1%) directly cited the AWaRe system in the wording of the indicator (Table S2). However, 445/773 indicators (57.6%) reflecting the contents and treatment recommendations of the AWaRe book were identified (Table S3). In total, 320/773 indicators (41.4%) were defined as ‘non-AWaRe indicators’ because they focused on: national/regional/local policies (203, 63.4%), settings (60, 18.8%) or infectious diseases (21, 6.6%) not included in the AWaRe book, patients with special conditions (19, 5.9%), laboratory tests (15, 4.7%) or therapies (2, 0.6%) not included in the AWaRe book (Table S4). The detailed list of indicators included in our review is available in Tables S2–S4.

### Reported method of indicator development

The majority of studies documented in Table 2 utilized a consensus methodology for the formulation of indicators. Most studies (*n* = 23), used a RAND/UCLA Appropriateness Method,^76^ 7 studies used a Delphi Technique procedure,^77,78^ 11 studies developed QIs through other consensus methods with a description of how consensus was obtained (e.g. multidisciplinary team agreement),^79^ 6 studies did not describe the consensus method used.

## Discussion

### Principal findings

We identified 773 indicators for appropriate antibiotic use of which only 1% were directly and 57.6% were indirectly related to the AWaRe system. Around 50% of infection-based indicators focused on RTIs, while for some serious infections (e.g. osteoarticular and abdominal infections) no indicators were identified. There was a similar distribution of indicators across primary and secondary care, with a high percentage of general indicators (45.9%) which can be used independently of the setting. Most of the indicators not included in the AWaRe book relate to the management of health services, health workers, and/or staff tasks, contrasting with those directly or indirectly related to the AWaRe system, which mostly focused on the consumption of antibiotics (frequency and/or volume of antibiotic use without reference to the indication) and antibiotic prescribing/dispensing. Among the latter, only 26 indicators included information on therapy review. 8.7% of indicators focused on the diagnostic process, and among them, 37.3% were based on the results of microbiological investigations.

### Comparison with the previous literature

Improving the quality of care and reducing avoidable harm requires reliable, valid and comparable data.^10^ Quality assessment leads to a steady improvement in antibiotic prescribing, allowing institutions to track their progress towards targets over time and to compare with other health facilities.^80–87^ In accordance with the literature,^30,63,88^ our review highlighted the increasing number of QIs for appropriate antibiotic use developed in recent decades, with considerable emphasis on RTIs. This finding could be due to the high prevalence of patients with RTIs and the relatively high percentage of antibiotic prescriptions for this condition both in primary and secondary care, despite the predominantly viral nature of RTIs.^89–91^ In recent years, the high rate of inappropriate antibiotic prescriptions in this patient category has resulted in RTIs becoming the focus of ASPs, especially in primary care in LMICs.^92^ Skin/soft tissue and intra-abdominal infections are also among the main indications for prescribing antibiotics in hospitals and ambulatory care,^89^ despite the almost total absence of indicators for these types of infections.^84,88^

To optimize antibiotic use, several aspects of care must be considered. A multi-faceted strategy based on the development of national/local guidelines, the allocation of adequate resources, and the creation of an experienced and competent team are key to responsible antibiotic use.^13,36,72,93,94^ Indicators focused on antibiotic prescribing and/or dispensing remain a fundamental tool to monitor appropriate antibiotic use. Among these, the review of therapy, closely linked to the performance of microbiological investigations, is a crucial aspect of the appropriate use of antibiotics. As highlighted by national guidelines,^77^ differences in local resistance patterns and antibiotic availability (or lack of availability) may prevent the use of the same class of antibiotics as empirical therapy worldwide. Nevertheless, switching from IV to oral therapy at an appropriate time and using pathogen-directed therapy as soon as possible are associated with a reduction in the length of hospital stay^95^ and antibiotic use.^96,97^

In our review, not surprisingly we identified only a few indicators directly citing the relatively new AWaRe system. In 2019, the monitoring and evaluation framework for the GAP on AMR provided a core set of indicators measurable by countries, including the use of the AWaRe system in monitoring national antibiotic consumption (Table 1).^4^ To date, no indicators that prioritize the quality, rather than the volume, of antibiotic use in alignment with the AWaRe book contents have been developed.

### Bias and limitations

This study has clear limitations. A formal systematic review was not conducted, and only English language publications were included, so some studies may have been missed. Secondly, only the MEDLINE database was searched, a limitation which was mitigated by screening the reference lists of all included articles and exploring the grey literature by including relevant websites.

### Next steps

Many countries are now implementing national action plans (NAPs) on AMR although at different stages of implementation,^13,98,99^ with the optimization of antibiotic use a key priority. Generating standardised, quality assured, globally comparable data is essential to the continuous improvement of ASPs and NAPs. QIs for antibiotic prescribing allow data to be collected on both the consumption and the quality of antibiotic care. The AWaRe book provides essential educational elements, including clinical diagnosis and treatment of the most frequent infections in health care and is a key instrument for ASPs.^100^ The introduction of disease-specific QIs based on the AWaRe system and book, both in therapeutic and diagnostic terms, could provide discrete and measurable elements of quality that could be used globally and be comparable between countries.^84^ Designed with the overarching goal of reducing the inappropriate use of antibiotics, the AWaRe book champions a targeted risk-based approach, advocating for ‘no antibiotic care’ when appropriate. At the core of its recommendations lies the emphasis on the appropriate use of the Access group antibiotics. Following the principles of the AWaRe system and stratifying total AMU by the AWaRe groups, allows overall monitoring of national and global progress towards a country-level target of at least 60% of total antibiotic consumption being Access group antibiotics, as outlined in the WHO 13th General Programme of Work.^101^

The small number of existing indicators related directly to the AWaRe system/book identified in this review suggests the next step is to develop new AWaRe QIs as essential tools to improve future antibiotic use.

Consensus techniques are fundamental and effective tools for quality improvement, enabling the evaluation and enhancement of different aspects of care where evidence is contested or not used appropriately. Most of the studies included in this review (50%) used the RAND/UCLA Appropriateness Method to develop new indicators. The Delphi Technique and RAND/UCLA Appropriateness Method are both widely used for the formulation of indicators, but the latter has been described as the only systematic method of combining expert opinion and evidence,^12^ resulting in widespread use^17,30,37,39,46,50,63^ and it is important to adhere to optimal use and reporting of feedback in a Delphi Technique.^78^

With this purpose, a Delphi Technique has been conducted with panellists across WHO regions and both Higher Income and Lower- and Middle-Income countries to assess the appropriateness and feasibility in local settings of indicators based on the findings of this review. This will be followed by a formal RAND/UCLA Appropriateness Method with leading international experts to assess the clarity, appropriateness, and feasibility of all the QIs globally, in all countries. Because indicators identified using a narrative literature review do not assess validity and the outcome of a consensus technique such as a Delphi Technique or RAND/UCLA Appropriateness Method provides only face validity,^11^ future research activities will then seek to validate and test the indicators using an indicator testing protocol including content validity, reliability and feasibility to underpin their potential purpose applied at both the local context and at a global level for quality assessment and improvement based on the WHO AWaRe system.^14,102^

## Conclusions

Being able to measure the quality of antibiotic prescribing is an essential prerequisite to promoting the appropriate use of antibiotics, reducing unnecessary prescribing, and mitigating antibiotic resistance. Despite the global awareness of the urgency of this issue and the efforts made so far, our review revealed the lack of discrete and dedicated QIs based on the WHO AWaRe system. These findings highlight the need to develop and test indicators based directly on the AWaRe system focused on their feasible integration and implementation into both local and national ASPs.

## Supplementary Material

dkae370_Supplementary_Data

## Supplementary data

Figure S1 and Tables S1–S4 are available as Supplementary data at *JAC* Online.
