# Supplementary material for: Identifying AWaRe indicators for appropriate antibiotic use: a narrative review
Source: J Antimicrob Chemother. 2024 Oct 18;79(12):3063–77. doi: 10.1093/jac/dkae370 (PMC11638856; doi:10.1093/jac/dkae370)
Supplement: dkae370_Supplementary_Data [file dkae370_supplementary_data.docx]

**Figure S1.** Medline search strategy.

"Anti-Bacterial Agents"[Mesh] OR

"Bacterial Infections*"[Mesh] OR

"antibiotic*" OR

"antibiotic agent*" OR

"anti-bacterial*" OR

“antimicrobial* OR

"antibiotic prescri*" OR

"antibiotic use" OR

"antibiotic treatment" OR

"antibiotic therapy" OR

“antimicrobial stewardship” OR

“antibiotic stewardship”

"Quality Indicators, Health Care"[Mesh] OR

“quality indicator” OR

“quantity metric*” OR

“activity indicator*” OR

“quality metric*” OR

“quantity indicator*” OR

“quality measure*” OR

“quality criteria” OR

“process indicator” OR

“outcome indicator” OR

“structure indicator” OR

“clinical indicator”

AND

**MeSH:** *Medical Subject Heading.*

**Table S1.** List of websites of national and international infectious disease societies and public health organisations screened.

| **Africa CDC:** <https://africacdc.org/> |
| --- |
| ***AHRQ’s NHQR (Agency for Healthcare Research and Quality):** <https://qualityindicators.ahrq.gov/> |
| **Alliance for the Prudent Use of Antibiotics (APUA)**: [www.apua.org/](http://www.apua.org/) |
| ***Australian Commission on Safety and Quality in Health Care:** <https://www.safetyandquality.gov.au/our-work/clinical-care-standards/antimicrobial-stewardship-clinical-care-standard/indicators#list-of-indicators> |
| ***BSAC**: <https://academic.oup.com/jacamr/article/1/2/dlz026/5554098?searchresult=1> |
| **Canadian Centre for Health and Safety in Agriculture:** [www.cchsa-ccssma.usask.ca/](http://www.cchsa-ccssma.usask.ca/) |
| **CDC:** <https://www.cdc.gov/antibiotic-use/core-elements/hospital.html> |
| **Chinese Center for Disease Control and Prevention:** <https://en.chinacdc.cn/> |
| ***CMS (Centers for Medicare & Medicaid Services - 2018 MIPS Quality Measures Related to Management of Adult ID):** <https://qpp.cms.gov/mips/explore-measures?tab=qualityMeasures&py=2018> |
| **European Center for Disease Control and Prevention (ECDC)**: [www.ecdc.europa.eu/](http://www.ecdc.europa.eu/) |
| **European Medicines Agency (EMA):** [www.ema.europa.eu/](http://www.ema.europa.eu/) |
| **European Society of Clinical Microbiology and Infectious Diseases (ESCMID)**: <https://www.escmid.org/> |
| **International Society of Infectious Diseases**: [www.isid.org/](http://www.isid.org/) |
| ***Israeli National Program for Quality Indicators in Community Healthcare (Surgical Site Infections):** <https://en.israelhealthindicators.org/> |
| **Korea Disease Control Prevention and Control Agency:** <http://www.kdca.go.kr/eng/> |
| ***NCQA - HEDIS (Healthcare Effectiveness Data and Information Set):** [HEDIS Measures and Technical Resources - NCQA](https://www.ncqa.org/hedis/measures/); <https://www.ncqa.org/hedis/measures/antibiotic-utilization/> |
| ***NHS England - CQUIN (NHS Commissioning for Quality and Innovation) Framework:** <https://www.england.nhs.uk/nhs-standard-contract/cquin/cquin-23-24/> |
| ***NICE**: <https://www.nice.org.uk/guidance/published?ndt=Quality%20standard&ps=9999> |
| ***OECD:** <https://www.oecd.org/els/health-systems/hcqi-primary-care.htm> |
| ***PAHO (Pan American Health Organization)**: <https://iris.paho.org/handle/10665.2/49645> |
| **RAND Corporation**: [www.rand.org/](http://www.rand.org/) |
| ***SAPG**: <https://www.sapg.scot/media/6994/sapg-opat-kpis-final.pdf>. <https://www.isdscotland.org/Health-Topics/Prescribing-and-Medicines/Publications/2014-10-14/2014-10-14-SAPG-Primary-Care-PI-2013-14-Report.pdf> |
| ***UKHSA (UK Health Security Agency):**  <https://fingertips.phe.org.uk/profile/amr-local-indicators> |
| **US Food and Drug Administration:** [www.fda.gov/](http://www.fda.gov/) |
| ***WHO M&E Framework**: <https://apps.who.int/iris/bitstream/handle/10665/325006/9789241515665-eng.pdf?sequence=1&isAllowed=y>  ***WHO TOOLKIT ASPs in LMICS**: <https://apps.who.int/iris/bitstream/handle/10665/329404/9789241515481-eng.pdf?sequence=1&isAllowed=y>  ***WHO/INRUD prescribing indicators**: <https://apps.who.int/iris/bitstream/handle/10665/60519/WHO_DAP_93.1.pdf> |
|  |
|  |
| **World Bank:** <https://www.worldbank.org/en/home> |

***This source provided indicators included in the final set.**

**Table S2.** Final set of quality indicators directly based on the AWaRe system included in our review.

| N | Indicator | Type | Setting | Target |
| --- | --- | --- | --- | --- |
|  | Proportion of Access antibiotics for systemic use, relative to total antibiotic consumption in DDD ^1^ | Clinical | General | Consumption of antibiotics/Prescription rate |
|  | Proportion of total antibiotic prescribing from the "Access" category of the WHO Essential Medicines List AWaRe index; by quarter and acute trust ^2^ | Clinical | General | Consumption of antibiotics/Prescription rate |
|  | Proportion of DDD in AWaRe and OTHER groups ^3^ | Clinical | General | Consumption of antibiotics/Prescription rate |
|  | Relative proportion of AWaRe (Access, Watch and Reserve) antibiotics for paediatric formulations ^1^ | Clinical | General | Consumption of antibiotics/Prescription rate |
|  | Percentage of adult and paediatric hospital patients receiving an antibiotic according to AWaRe categories ^1^ | Clinical | General | Consumption of antibiotics/Prescription rate |
|  | Proportion of total antibiotic prescribing from the “Watch” category of the WHO Essential Medicines List AWaRe index ^2^ | Clinical | General | Consumption of antibiotics/Prescription rate |
|  | Proportion of total antibiotic prescribing from the “Reserve” category of the WHO Essential Medicines List AWaRe index ^2^ | Clinical | General | Consumption of antibiotics/Prescription rate |
|  | List of “Reserve” antibiotics with authorisation system for delivery available ^4^ | Organisational | Generic | Management |

DDD = Defined Daily Doses

**Table S3**. Final set of quality indicators and quantity metrics indirectly related to the AWaRe system included in our review.

| N | Indicator | Type of infection | Type | Setting | Target |
| --- | --- | --- | --- | --- | --- |
|  | Percentage of patients aged between 18 and 75 years with acute bronchitis/bronchiolitis prescribed antibacterials for systemic use ^5^ | RTIs | Clinical | Primary health care | Consumption of antibiotics/Prescription rate |
|  | Percentage of patients aged between 18 and 75 years with acute bronchitis/bronchiolitis prescribed antibacterials for systemic use receiving the recommended antibacterials ^5^ | RTIs | Clinical | Primary health care | Antibiotic prescribing/dispensing |
|  | Percentage of patients aged between 18 and 75 years with acute bronchitis/bronchiolitis prescribed antibacterials for systemic use receiving quinolones ^5^ | RTIs | Clinical | Primary health care | Antibiotic prescribing/dispensing |
|  | Patients with acute exacerbation of chronic obstructive pulmonary disease (COPD). Number of patients treated with antibiotics ^6^ | RTIs | Clinical | Primary health care | Consumption of antibiotics/Prescription rate |
|  | Patients with acute exacerbation of chronic obstructive pulmonary disease (COPD).   Number of patients not fulfilling all the Anthonisen criteria 2 treated with antibiotics ^6^ | RTIs | Clinical | Primary health care | Antibiotic prescribing/dispensing |
|  | Patients with acute exacerbation of COPD. Number of patients with acute exacerbation of dyspnea, coughing and/or expectoration greater than the daily variation / Number of patients ^6^ | RTIs | Clinical | Primary health care | Diagnostic process |
|  | Patients with acute bronchitis.  Number of patients with purulent expectorate treated with antibiotics / Number of patients with purulent expectorate ^6^ | RTIs | Clinical | Primary health care | Antibiotic prescribing/dispensing |
|  | Patients with acute exacerbation of COPD. Number of patients fulfilling 2–3 Anthonisen criteria treated with antibiotics / Number of patients fulfilling 2–3 Anthonisen criteria ^6^ | RTIs | Clinical | Primary health care | Diagnostic process |
|  | Patients with acute exacerbation of severe (class C–D) COPD.  Number of patients treated with antibiotics / Number of patients ^6^ | RTIs | Clinical | Primary health care | Diagnostic process |
|  | Patients with acute exacerbation of mild–moderate (class A–B) COPD. Number of patients with a CRP test <10 mg/l and/or absence of fever fulfilling less than two Anthonisen criteria treated with antibiotics / Number of patients with a CRP test <10 mg/l and/or absence of fever fulfilling less than two Anthonisen criteria ^6^ | RTIs | Clinical | Primary health care | Diagnostic process |
|  | Patients with acute bronchitis.  Number of patients treated with penicillin V / Number of patients treated with antibiotics ^6^ | RTIs | Clinical | Primary health care | Antibiotic prescribing/dispensing |
|  | Patients with acute bronchitis. Number of patients treated with amoxicillin ± clavulanic acid / Number of patients treated with antibiotics ^6^ | RTIs | Clinical | Primary health care | Antibiotic prescribing/dispensing |
|  | Patients with acute bronchitis. Number of patients without known penicillin allergy treated with macrolides / Number of patients treated with macrolides ^6^ | RTIs | Clinical | Primary health care | Antibiotic prescribing/dispensing |
|  | Patients with acute exacerbation of COPD. Number of patients without known penicillin allergy treated with quinolones / Number of patients treated with quinolones ^6^ | RTIs | Clinical | Primary health care | Antibiotic prescribing/dispensing |
|  | Proportion of patients (no relevant comorbidities) presenting with acute cough that should be prescribed oral antibiotics ^7^ | RTIs | Clinical | Primary health care | Antibiotic prescribing/dispensing |
|  | Proportion of patients (no relevant comorbidities) presenting with acute bronchitis that should be prescribed oral antibiotics ^7^ | RTIs | Clinical | Primary health care | Antibiotic prescribing/dispensing |
|  | Proportion of patients presenting with an acute exacerbation of their COPD that should be prescribed oral antibiotics ^7^ | RTIs | Clinical | Primary health care | Antibiotic prescribing/dispensing |
|  | Prescribe antibiotic therapy for exacerbations of chronic bronchitis or chronic obstructive pulmonary disease care only when indicated ^8^ | RTIs | Clinical | Primary health care | Antibiotic prescribing/dispensing |
|  | Antibiotics should not be prescribed for (most) viral infections or self-limiting bacterial infections (e.g. acute bronchitis, influenza, acute otitis media in patients > 2 years of age) ^9^ | RTIs | Clinical | General | Antibiotic prescribing/dispensing |
|  | Percentage of patients older than 1 year with acute upper respiratory infection prescribed antibacterials for systemic use ^10^ | RTIs | Clinical | Primary health care | Consumption of antibiotics/Prescription rate |
|  | Percentage of patients older than 1 year with acute upper respiratory infection prescribed antibacterials for systemic use receiving the recommended antibacterials ^10^ | RTIs | Clinical | Primary health care | Antibiotic prescribing/dispensing |
|  | Percentage of patients older than 1 year with acute upper respiratory infection prescribed antibacterials for systemic use receiving quinolones ^10^ | RTIs | Clinical | Primary health care | Antibiotic prescribing/dispensing |
|  | Acute upper respiratory infections and bronchitis should not be treated with antibiotics within the first 3 days, unless there is documented indication for treatment ^10, 11^ | RTIs | Clinical | Primary health care | Antibiotic prescribing/dispensing |
|  | Appropriate treatment for children with URTIs, i.e. of children 3 months to 18 years of age, with URTIs not prescribed antibiotics on or three days after the episode date ^12^ | RTIs | Clinical | Primary health care | Antibiotic prescribing/dispensing |
|  | Percentage of children 3 months-18 years of age who were diagnosed with upper respiratory infection (URI) and were not dispensed an antibiotic prescription on or three days after the episode ^5^ | RTIs | Clinical | Primary health care | Antibiotic prescribing/dispensing |
|  | Percentage of patients older than 1 year with acute tonsillitis prescribed antibacterials for systemic use ^10^ | RTIs | Clinical | Primary health care | Consumption of antibiotics/Prescription rate |
|  | Percentage of patients older than 1 year with acute tonsillitis prescribed antibacterials for systemic use receiving the recommended antibacterials ^10^ | RTIs | Clinical | Primary health care | Antibiotic prescribing/dispensing |
|  | Percentage of patients older than 1 year with acute tonsillitis prescribed antibacterials for systemic use receiving quinolones ^10^ | RTIs | Clinical | Primary health care | Antibiotic prescribing/dispensing |
|  | Swabbing the throat and testing for Group A streptococcal (GAS) pharyngitis by RADT and/or culture should be performed ^13^ | RTIs | Clinical | Primary health care | Diagnostic process |
|  | In children and adolescents, a negative rapid antigen detection tests (RADT) test should be backed up by a throat culture ^13^ | RTIs | Clinical | Primary health care | Diagnostic process |
|  | In children and adolescents, a positive RADT test should not be backed up by a throat culture ^13^ | RTIs | Clinical | Primary health care | Diagnostic process |
|  | Patients with acute GAS pharyngitis should be treated with an appropriate antibiotic ^13^ | RTIs | Clinical | Primary health care | Antibiotic prescribing/dispensing |
|  | Patients with acute tonsillitis/pharyngitis. Number of patients examined with a StrepA test ^14^ | RTIs | Clinical | Primary health care | Diagnostic process |
|  | Patients with acute tonsillitis/pharyngitis.  Number of patients treated with antibiotics without StrepA test ^14^ | RTIs | Clinical | Primary health care | Diagnostic process |
|  | Patients with acute tonsillitis/pharyngitis. Number of patients treated with antibiotics with negative StrepA test ^14^ | RTIs | Clinical | Primary health care | Diagnostic process |
|  | Outpatients with an acute tonsillitis/pharyngitis and positive group A streptococcal diagnostic test should be treated with antibiotics ^9^ | RTIs | Clinical | Primary health care | Antibiotic prescribing/dispensing |
|  | Antibiotics for an acute tonsillitis/pharyngitis should be withheld, discontinued or not prescribed if an outpatient presents a diagnostic test (rapid antigen test or throat culture) negative for group A streptococci ^9^ | RTIs | Clinical | Primary health care | Antibiotic prescribing/dispensing |
|  | Patients with acute pharyngotonsillitis. Number of patients fulfilling 2–3 modified Centor criteria examined with a StrepA test / Number of patients fulfilling 2–3 modified Centor criteria ^6^ | RTIs | Clinical | Primary health care | Diagnostic process |
|  | Patients with acute pharyngotonsillitis. Number of patients fulfilling 0-1 modified Centor criteria examined with a StrepA test / Number of patients fulfilling 0-1 modified Centor criteria ^6^ | RTIs | Clinical | Primary health care | Diagnostic process |
|  | Patients with acute pharyngotonsillitis. Number of patients with a positive StrepA test treated with antibiotics / Number of patients with a positive StrepA test ^6^ | RTIs | Clinical | Primary health care | Diagnostic process |
|  | Patients with acute pharyngotonsillitis. Number of patients fulfilling 0–1 modified Centor criterion treated with antibiotics / Number of patients fulfilling 0–1 modified Centor criterion ^6^ | RTIs | Clinical | Primary health care | Diagnostic process |
|  | Patients with acute pharyngotonsillitis. Number of generally affected patients fulfilling 4–5 modified Centor criteria treated with antibiotics / Number of generally affected patients fulfilling 4–5 modified Centor criteria ^6^ | RTIs | Clinical | Primary health care | Diagnostic process |
|  | Patients with acute pharyngotonsillitis. Number of patients treated with penicillin V / Number of patients treated with antibiotics ^6^ | RTIs | Clinical | Primary health care | Antibiotic prescribing/dispensing |
|  | Patients with acute pharyngotonsillitis. Number of patients without known penicillin allergy treated with macrolides / Number of patients treated with macrolide ^6^ | RTIs | Clinical | Primary health care | Antibiotic prescribing/dispensing |
|  | Proportion of patients (no relevant comorbidities) that should be prescribed oral antibiotics when presenting with acute sore throat ^7^ | RTIs | Clinical | Primary health care | Antibiotic prescribing/dispensing |
|  | Acute sore throat. Restrictive prescribing ^15^ | RTIs | Clinical | Primary health care | Antibiotic prescribing/dispensing |
|  | Acute sore throat, acute tonsillitis. First choice feniticillin, phenoxymethylpenicillin ^15^ | RTIs | Clinical | Primary health care | Antibiotic prescribing/dispensing |
|  | Clinicians should not administer or prescribe perioperative antibiotics to children undergoing tonsillectomy ^13^ | RTIs | Clinical | Hospital facility | Antibiotic prescribing/dispensing |
|  | Percentage of patients older than 18 years with acute/chronic sinusitis prescribed antibacterials for systemic use receiving the recommended antibacterials ^10^ | RTIs | Clinical | Primary health care | Antibiotic prescribing/dispensing |
|  | Percentage of patients older than 18 years with acute/chronic sinusitis prescribed antibacterials for systemic use receiving quinolones ^10^ | RTIs | Clinical | Primary health care | Antibiotic prescribing/dispensing |
|  | Antibiotics may be prescribed for acute bacterial rhinosinusitis (ABRS) if disease severely impacts quality of life (QOL)/productivity, the condition worsens, the patient is unable to follow- up, and/or the patient’s conditions fail to improve by 7 days after ABRS diagnosis ^16^ | RTIs | Clinical | Primary health care | Diagnostic process |
|  | Amoxicillin for 5–10 days should be used as first line antibiotic therapy line antibiotic therapy for acute bacterial rhinosinusitis (ABRS) ^16^ | RTIs | Clinical | Primary health care | Antibiotic prescribing/dispensing |
|  | Sinusitis.  Increase of general symptoms, risk factors for complications, no decrease of symptoms after 2 weeks / amoxicillin or doxycycline ^17^ | RTIs | Clinical | Primary health care | Antibiotic prescribing/dispensing |
|  | Patients with acute sinusitis.  Number of patients with symptoms for less than 1 week ^14^ | RTIs | Clinical | Primary health care | Diagnostic process |
|  | Patients with acute sinusitis.  Number of patients examined with a CRP test ^14^ | RTIs | Clinical | Primary health care | Diagnostic process |
|  | Patients with acute sinusitis. Number of patients treated with antibiotics without a diagnostic test ^14^ | RTIs | Clinical | Primary health care | Diagnostic process |
|  | Patients with acute rhinosinusitis.  Number of patients with >10 days symptom duration or increasing symptoms after 5 days / Number of patients ^6^ | RTIs | Clinical | Primary health care | Diagnostic process |
|  | Patients with acute rhinosinusitis. Number of patients treated with antibiotics / Number of patients ^6^ | RTIs | Clinical | Primary health care | Consumption of antibiotics/Prescription rate |
|  | Patients with acute rhinosinusitis. Number of patients with a CRP test <10 mg/l treated with antibiotics / Number of patients with a CRP test <10 mg/l ^6^ | RTIs | Clinical | Primary health care | Diagnostic process |
|  | Patients with acute rhinosinusitis.  Number of patients fulfilling less than three diagnostic criteria treated with antibiotics / Number of patients fulfilling less than three diagnostic criteria ^6^ | RTIs | Clinical | Primary health care | Diagnostic process |
|  | Patients with acute rhinosinusitis. Number of patients fulfilling three or more diagnostic criteria treated with antibiotics / Number of patients fulfilling three or more diagnostic criteria ^6^ | RTIs | Clinical | Primary health care | Diagnostic process |
|  | Patients with acute rhinosinusitis. Number of patients with <5 days symptom duration treated with antibiotics / Number of patients with <5 days symptom duration ^6^ | RTIs | Clinical | Primary health care | Diagnostic process |
|  | Patients with acute rhinosinusitis. Number of patients treated with penicillin V / Number of patients treated with antibiotics ^6^ | RTIs | Clinical | Primary health care | Antibiotic prescribing/dispensing |
|  | Patients with acute rhinosinusitis. Number of patients without known penicillin allergy treated with macrolides / Number of patients treated with macrolides ^6^ | RTIs | Clinical | Primary health care | Antibiotic prescribing/dispensing |
|  | Proportion of patients (no relevant comorbidities) that should be prescribed oral antibiotics when presenting with acute rhinosinusitis ^7^ | RTIs | Clinical | Primary health care | Antibiotic prescribing/dispensing |
|  | Percentage of patients, aged 18 years and older, with a diagnosis of acute sinusitis who were prescribed an antibiotic within 10 days after onset of symptoms ^5^ | RTIs | Clinical | Primary health care | Diagnostic process |
|  | Percentage of patients older than 2 years with acute otitis media/myringitis prescribed antibacterials for systemic use ^10^ | RTIs | Clinical | Primary health care | Consumption of antibiotics/Prescription rate |
|  | Percentage of patients older than 2 years with acute otitis media/myringitis prescribed antibacterials for systemic use receiving the recommended antibacterials ^10^ | RTIs | Clinical | Primary health care | Antibiotic prescribing/dispensing |
|  | Percentage of patients older than 2 years with acute otitis media/myringitis prescribed antibacterials for systemic use receiving quinolones ^10^ | RTIs | Clinical | Primary health care | Antibiotic prescribing/dispensing |
|  | Otitis media acuta. Antibiotics when - increase in general signs as decreased alertness or decrease in fluid consumption - risk factors for complications - no improvement after 3 days - child: < 2 years with otitis media at two sites ^17^ | RTIs | Clinical | Primary health care | Diagnostic process |
|  | Patients with acute otitis media (AOM).  Number of patients > 2 years with symptoms for less than 3 days ^14^ | RTIs | Clinical | Primary health care | Diagnostic process |
|  | Patients with acute otitis media (AOM). Number of patients < 2 years treated with antibiotics ^14^ | RTIs | Clinical | Primary health care | Consumption of antibiotics/Prescription rate |
|  | Patients with acute otitis media (AOM). Number of patients > 2 years with less than 3 days of symptoms of AOM treated with antibiotics ^14^ | RTIs | Clinical | Primary health care | Consumption of antibiotics/Prescription rate |
|  | Patients with acute otitis media.  Number of patients >6 months fulfilling one or more diagnostic criteria and reduced mobility of the eardrum / Number of patients >6 months ^6^ | RTIs | Clinical | Primary health care | Diagnostic process |
|  | Patients with acute otitis media. Number of patients >6 months with an evaluation of the eardrum mobility / Number of patients >6 months ^6^ | RTIs | Clinical | Primary health care | Diagnostic process |
|  | Patients with acute otitis media. Number of patients <6 months treated with antibiotics / Number of patients <6 months ^6^ | RTIs | Clinical | Primary health care | Consumption of antibiotics/Prescription rate |
|  | Patients with acute otitis media. Number of patients >6 months with no signs of fluid in the middle ear treated with antibiotics / Number of patients >6 months with no signs of fluid in the middle ear ^6^ | RTIs | Clinical | Primary health care | Diagnostic process |
|  | Patients with acute otitis media. Number of patients >6 months with 3 days of acute ear pain and no signs of fluid in the middle ear treated with antibiotics / Number of patients >6 months with 3 days of acute ear pain and no signs of fluid in the middle ear ^6^ | RTIs | Clinical | Primary health care | Diagnostic process |
|  | Patients with acute otitis media. Number of patients treated with penicillin V / Number of patients treated with antibiotics ^6^ | RTIs | Clinical | Primary health care | Antibiotic prescribing/dispensing |
|  | Patients with acute otitis. Number of patients treated with amoxicillin ± clavulanic acid / Number of patients treated with antibiotics ^6^ | RTIs | Clinical | Primary health care | Antibiotic prescribing/dispensing |
|  | Patients with acute otitis media. Number of patients without known penicillin allergy treated with macrolides / Number of patients treated with macrolides ^6^ | RTIs | Clinical | Primary health care | Antibiotic prescribing/dispensing |
|  | Proportion of patients (aged 6 months to 2 years) presenting with acute otitis media and not having bilateral disease and/or otorrhoea that should be prescribed oral antibiotics ^7^ | RTIs | Clinical | Primary health care | Diagnostic process |
|  | Proportion of patients (aged 2 years to 18 years) presenting with acute otitis media and not having bilateral disease and/or otorrhoea that should be prescribed oral antibiotics ^7^ | RTIs | Clinical | Primary health care | Diagnostic process |
|  | Percentage of patients aged between 18 and 65 years with pneumonia prescribed antibacterials for systemic use receiving the recommended antibacterials ^10^ | RTIs | Clinical | Hospital facility, Primary health care | Antibiotic prescribing/dispensing |
|  | Percentage of patients aged between 18 and 65 years with pneumonia prescribed antibacterials for systemic use receiving quinolones ^10^ | RTIs | Clinical | Hospital facility, Primary health care | Antibiotic prescribing/dispensing |
|  | Pneumonia. Children amoxicillin; second choice azitromycine.  Adults doxycyclin; second choice amoxicillin; third choice erythromycin ^17^ | RTIs | Clinical | Hospital facility, Primary health care | Antibiotic prescribing/dispensing |
|  | Patients with pneumonia. Number of patients fulfilling less than two diagnostic criteria / Number of patients ^6^ | RTIs | Clinical | Hospital facility, Primary health care | Diagnostic process |
|  | Patients with pneumonia. Number of patients examined with a CRP test / Number of patients ^6^ | RTIs | Clinical | Hospital facility | Diagnostic process |
|  | Patients with pneumonia. Number of patients with a CRP test <20 mg/l / Number of patients ^6^ | RTIs | Clinical | Hospital facility | Diagnostic process |
|  | Patients with pneumonia.  Number of patients examined with an X-ray of thorax / Number of patients ^6^ | RTIs | Clinical | Hospital facility | Diagnostic process |
|  | Patients with pneumonia. Number of patients treated with antibiotics / Number of patients ^6^ | RTIs | Clinical | Hospital facility, Primary health care | Consumption of antibiotics/Prescription rate |
|  | Patients with pneumonia. Number of patients <65 years fulfilling less than diagnostic criteria treated with antibiotics / Number of patients <65 years fulfilling less than two diagnostic criteria ^6^ | RTIs | Clinical | Hospital facility | Diagnostic process |
|  | Patients with pneumonia. Number of patients treated with penicillin V / Number of patients treated with antibiotics ^6^ | RTIs | Clinical | Hospital facility, Primary health care | Antibiotic prescribing/dispensing |
|  | Patients with pneumonia.  Number of patients without known penicillin allergy treated with macrolides / Number of patients treated with macrolides ^6^ | RTIs | Clinical | Hospital facility, Primary health care | Antibiotic prescribing/dispensing |
|  | Treatment of community acquired pneumonia in line with BTS care bundle ^18^ | RTIs | Clinical | Hospital facility, Primary health care | Antibiotic prescribing/dispensing |
|  | Proportion of adults with low‑severity community‑acquired pneumonia who receive a 5‑day maximum course of a single antibiotic ^19^ | RTIs | Clinical | Primary health care | Antibiotic prescribing/dispensing |
|  | Proportion of hospital admissions of community‑acquired pneumonia in adults at which antibiotic therapy is started within 4 hours of presentation ^19^ | RTIs | Clinical | Hospital facility | Antibiotic prescribing/dispensing |
|  | Pneumonia Mortality Rate ^20^ | RTIs | Clinical | Hospital facility | Outcome |
|  | Community Acquired Pneumonia Admission Rate ^20^ | RTIs | Clinical | Hospital facility | Outcome |
|  | Length of therapy is five days for uncomplicated CAP (CURB score ≤ 2) ^21^ | RTIs | Clinical | Primary health care | Antibiotic prescribing/dispensing |
|  | CAP.  Stop antibiotic therapy if no fever for 3 consecutive days ^3^ | RTIs | Clinical | Primary health care, Hospital facility | Antibiotic prescribing/dispensing |
|  | CAP in children.  Proportion of antibiotic use ^22^ | RTIs | Clinical | Hospital facility, Primary health care | Consumption of antibiotics/Prescription rate |
|  | CAP in children. Proportion of broad-spectrum antibiotic use after pathogen identification ^22^ | RTIs | Clinical | Hospital facility, Primary health care | Consumption of antibiotics/Prescription rate |
|  | CAP in children.  Proportion of macrolide antibiotic use ^22^ | RTIs | Clinical | Hospital facility, Primary health care | Consumption of antibiotics/Prescription rate |
|  | CAP in children. Proportion of antibiotic combination therapy ^22^ | RTIs | Clinical | Hospital facility, Primary health care | Consumption of antibiotics/Prescription rate |
|  | CAP in children. Proportion of the combined use of macrolide and b-lactaman antibiotics ^22^ | RTIs | Clinical | Hospital facility, Primary health care | Consumption of antibiotics/Prescription rate |
|  | CAP in children. Proportion of third-generation cephalosporin antibiotic use ^22^ | RTIs | Clinical | Hospital facility, Primary health care | Consumption of antibiotics/Prescription rate |
|  | CAP in children.  Microbiological examination rate of children with CAP who were receiving antibiotics ^22^ | RTIs | Clinical | Hospital facility | Diagnostic process |
|  | CAP in children. Antibacterial use density ^22^ | RTIs | Clinical | Hospital facility, Primary health care | Consumption of antibiotics/Prescription rate |
|  | CAP in children. Proportion of antibiotics administered intravenously ^22^ | RTIs | Clinical | Hospital facility | Antibiotic prescribing/dispensing |
|  | CAP in children. Proportion of sequential therapy ^22^ | RTIs | Clinical | Hospital facility, Primary health care | Consumption of antibiotics/Prescription rate |
|  | CAP in children. Average number of days of antibiotic treatment ^22^ | RTIs | Clinical | Hospital facility, Primary health care | Antibiotic prescribing/dispensing |
|  | CAP in children.  Average time of antibiotic treatment for children with pleural effusion or empyema ^22^ | RTIs | Clinical | Hospital facility | Consumption of antibiotics/Prescription rate |
|  | CAP. Obtain sputum sample and start antibiotic therapy in the emergency department ^21^ | RTIs | Organisational | Hospital facility | Management |
|  | CAP. Urine antigen testing for Legionella species on clinical suspicion ^8^ | RTIs | Organisational | Hospital facility | Diagnostic process |
|  | CAP. Duration of therapy no longer than 7 days (patients on general ward) ^23^ | RTIs | Organisational | Hospital facility | Antibiotic prescribing/dispensing |
|  | HAP. Duration of therapy no longer than 10 days ^23^ | RTIs | Organisational | Hospital facility | Antibiotic prescribing/dispensing |
|  | CAP. Combination therapy, if any, no longer than three days (patients on normal wards only) ^23^ | RTIs | Organisational | Hospital facility | Antibiotic prescribing/dispensing |
|  | Patients with acute lower respiratory tract infections.  Number of patients examined with a CRP test ^14^ | RTIs | Clinical | Hospital facility | Diagnostic process |
|  | Patients with acute lower respiratory tract infections. Number of patients examined with an X-ray of thorax ^14^ | RTIs | Clinical | Hospital facility | Diagnostic process |
|  | Patients with acute lower respiratory tract infections. Number of patients not examined with either a CRP test or X-ray of thorax ^14^ | RTIs | Clinical | Hospital facility | Diagnostic process |
|  | Patients with acute lower respiratory tract infections. Number of patients treated with antibiotics ^14^ | RTIs | Clinical | Hospital facility, Primary health care | Consumption of antibiotics/Prescription rate |
|  | Patients with acute lower respiratory tract infections.  Number of patients treated with antibiotics without a preceding CRP test or X-ray of thorax ^14^ | RTIs | Clinical | Hospital facility | Diagnostic process |
|  | Patients with acute lower respiratory tract infections. Number of patients treated with antibiotics with a CRP test < 20 mg/l ^14^ | RTIs | Clinical | Hospital facility | Diagnostic process |
|  | Patients whom the doctor suspects have a lower RTI. Number of patients seen on the same day / Number of patients ^6^ | RTIs | Clinical | Hospital facility, Primary health care | Diagnostic process |
|  | Percentage of female patients older than 18 years with cystitis/other urinary infection prescribed antibacterials for systemic use ^10^ | UTIs | Clinical | Hospital facility, Primary health care | Consumption of antibiotics/Prescription rate |
|  | Percentage of female patients older than 18 years with cystitis/other urinary infection prescribed antibacterials for systemic use receiving the recommended antibacterials ^10^ | UTIs | Clinical | Hospital facility, Primary health care | Antibiotic prescribing/dispensing |
|  | Percentage of female patients older than 18 years with cystitis/other urinary infection prescribed antibacterials for systemic use receiving quinolones ^10^ | UTIs | Clinical | Hospital facility, Primary health care | Antibiotic prescribing/dispensing |
|  | Complicated UTI with signs of tissue invasion, all boys < 13 years, girls < 5 years and all patients with known abnormalities to the kidneys or the urinary tract or having a permanent catheter: augmentin;  second choice: cotrimoxazol or fluorchinolon ^17^ | UTIs | Clinical | Hospital facility | Antibiotic prescribing/dispensing |
|  | Complicated UTI without signs of tissue invasion, all man, all pregnant woman, all girls between 5 and 12 and diabetics: nitrofurantoin; when allergic: trimethoprim ^17^ | UTIs | Clinical | Hospital facility | Antibiotic prescribing/dispensing |
|  | Proportion of women (non-pregnant, aged 14+, no relevant comorbidities) that should be prescribed oral antibiotics when actually having a bacterial urinary tract infection ^7^ | UTIs | Clinical | Hospital facility, Primary health care | Antibiotic prescribing/dispensing |
|  | Uncomplicated urinary tract infections (>12 years). First choice: trimethoprim or nitrofurantoin ^15^ | UTIs | Clinical | Primary health care | Antibiotic prescribing/dispensing |
|  | Percentage of antibiotic prescriptions for lower UTI in older people meeting NICE NG109 guidance and UKHSA Diagnosis of UTI guidance in terms of diagnosis and treatment; by quarter ^2^ | UTIs | Clinical | Primary health care | Antibiotic prescribing/dispensing |
|  | Appropriate antibiotic prescribing for UTI in adults aged 16+ ^18^ | UTIs | Clinical | Hospital facility, Primary health care | Antibiotic prescribing/dispensing |
|  | Proportion of episodes of suspected UTI in women aged under 65 with signs and symptoms documented in the patient's records ^19^ | UTIs | Clinical | Hospital facility, Primary health care | Diagnostic process |
|  | Proportion of women aged under 65 years diagnosed with a UTI who have 2 or more key urinary symptoms and no other excluding causes or warning signs ^19^ | UTIs | Clinical | Hospital facility, Primary health care | Diagnostic process |
|  | Prescription rates for antibiotics used for lower UTIs (such as trimethoprim, nitrofurantoin, fosfomycin or pivmecillinam) ^19^ | UTIs | Clinical | Primary health care | Consumption of antibiotics/Prescription rate |
|  | Antibiotic prescription rates for adults with indwelling urinary catheters ^19^ | UTIs | Clinical | Hospital facility, Primary health care | Consumption of antibiotics/Prescription rate |
|  | Proportion of episodes of suspected UTI in men and non-pregnant women with signs and symptoms documented in the patient's records ^19^ | UTIs | Clinical | Hospital facility, Primary health care | Diagnostic process |
|  | Proportion of episodes of asymptomatic bacteriuria in men and non-pregnant women treated with antibiotics ^19^ | UTIs | Clinical | Primary health care | Consumption of antibiotics/Prescription rate |
|  | Antimicrobial prescribing rates for men and non-pregnant women with asymptomatic bacteriuria ^19^ | UTIs | Clinical | Primary health care | Consumption of antibiotics/Prescription rate |
|  | Proportion of antibiotic courses prescribed for a 3-day duration for episodes of uncomplicated lower UTI in non-pregnant women ^19^ | UTIs | Clinical | Primary health care | Antibiotic prescribing/dispensing |
|  | Proportion of antibiotic courses prescribed for a 7-day duration for episodes of uncomplicated lower UTI in men ^19^ | UTIs | Clinical | Primary health care | Antibiotic prescribing/dispensing |
|  | Proportion of antibiotic courses prescribed for a 7-day duration for episodes of uncomplicated lower UTI in pregnant women ^19^ | UTIs | Clinical | Primary health care | Antibiotic prescribing/dispensing |
|  | Average duration of antibiotic treatment for UTI ^19^ | UTIs | Clinical | Hospital facility, Primary health care | Antibiotic prescribing/dispensing |
|  | Urinary Tract Infection Admission Rate (adults) ^20^ | UTIs | Clinical | Hospital facility | Outcome |
|  | Urinary Tract Infection Admission Rate (children) ^20^ | UTIs | Clinical | Hospital facility | Outcome |
|  | Start IV antibiotics in pregnant women with pyelonephritis ^24^ | UTIs | Clinical | Hospital facility | Antibiotic prescribing/dispensing |
|  | UTI.  Optimal duration of antibiotic therapy from 5-7 days ^8^ | UTIs | Clinical | Primary health care | Antibiotic prescribing/dispensing |
|  | Appropriate antibiotic prescription.  Kenian guideline recommended antibiotic (Nitrofurantoin 100 mg bid for 57 days)– OR– a reason why an alternative was chosen (e.g. ciprofloxacin for pyelonephritis, TMP-SMX due to previous GI intolerance with nitrofurantoin, or nausea in pregnancy, etc.) ^25^ | UTIs | Organisational | Primary health care | Antibiotic prescribing/dispensing |
|  | Duration of pyelonephritis therapy not longer than 10 days (patients on general ward) ^23^ | UTIs | Organisational | Hospital facility | Antibiotic prescribing/dispensing |
|  | Documented positive urine culture (significant bacteriuria, no mixed flora) ^23^ | UTIs | Organisational | Generic | Management |
|  | No antimicrobials for asymptomatic, catheter- associated bacteriuria ^23^ | UTIs | Organisational | Hospital facility, Primary health care | Antibiotic prescribing/dispensing |
|  | Oral antimicrobial drugs initiated not later than day 5 (pyelonephritis, patients on normal wards only) ^23^ | UTIs | Organisational | Hospital facility | Antibiotic prescribing/dispensing |
|  | Proportion of episodes of suspected catheter-associated UTI in adults where a urine sample is sent to laboratories for culture and sensitivity testing ^19^ | UTIs | Clinical | Hospital facility, Primary health care | Diagnostic process |
|  | cUTI.  Duration of treatment for at least 10 days (in accordance with national guideline) ^24^ | UTIs | Organisational | Hospital facility | Antibiotic prescribing/dispensing |
|  | Does your facility measure the number of antimicrobial prescriptions that are consistent with the local treatment recommendations for either urinary tract infection (UTI) or community-acquired pneumonia (CAP)? ^26^ | Other | Organisational | Hospital facility, Primary health care | Antibiotic prescribing/dispensing |
|  | Conservable days of therapy among certain patients (among patients who received a diagnosis of community-acquired pneumonia, skin or soft-tissue infection, sepsis, or blood stream infection) ^27^ | Other | Clinical | General | Antibiotic prescribing/dispensing |
|  | Antibiotics should be prescribed for (most) bacterial infections (e.g. acute pneumonia, urinary tract infections) ^9,11^ | Other | Clinical | General | Antibiotic prescribing/dispensing |
|  | Unplanned hospital readmission within 30 days after hospital discharge (among patients who received a diagnosis of community-acquired pneumonia, skin or soft-tissue infection, sepsis, or bloodstream infection) ^27^ | Other | Clinical | Hospital facility | Outcome |
|  | Does your patient have an infection that may require special consideration?  Infections that may require special consideration include: Deep-seated infection; Infection requiring high tissue concentration; Infection requiring prolonged IV therapy; Critical infection with high risk of mortality.  Specific infections for special consideration are: Bloodstream infection, Empyema, Endocarditis, Meningitis, Osteomyelitis, Severe or necrotising soft tissue infections, Septic arthritis, Undrained abscess. ^28^ | Other | Clinical | General | Antibiotic prescribing/dispensing |
|  | Rates of admission to critical care for people with sepsis ^19^ | Bloodstream infections | Clinical | Hospital facility | Outcome |
|  | Rates of in-hospital mortality for people with sepsis ^19^ | Bloodstream infections | Clinical | Hospital facility | Outcome |
|  | Proportion of people with suspected sepsis in acute hospital settings and at least 1 of the criteria indicating high risk of severe illness or death from sepsis who receive the first dose of intravenous antibiotics within 1 hour of risk being stratified ^19^ | Bloodstream infections | Clinical | Hospital facility | Antibiotic prescribing/dispensing |
|  | Rates of 28-day all-cause mortality in people with sepsis ^19^ | Bloodstream infections | Clinical | Hospital facility | Outcome |
|  | Incidence of vascular access device-related bloodstream infection ^19^ | Bloodstream infections | Clinical | Hospital facility | Outcome |
|  | Proportion of children and young people with suspected bacterial meningitis or meningococcal septicaemia who receive intravenous or intraosseous antibiotics within an hour of arrival at hospital ^19^ | Bloodstream infections | Clinical | Hospital facility | Antibiotic prescribing/dispensing |
|  | Postoperative Sepsis Rate (adults) ^20^ | Bloodstream infections | Clinical | Hospital facility | Outcome |
|  | Percentage of patients with sepsis due to MSSA bacteremia who received beta-lactam antibiotic (eg, nafcillin, oxacillin, or cefazolin) as definitive therapy ^5^ | Bloodstream infections | Clinical | Hospital facility | Antibiotic prescribing/dispensing |
|  | CRBSI rate in neonatology ^29^ | Bloodstream infections | Clinical | Hospital facility | Outcome |
|  | CRBSI rate in premature neonates ^29^ | Bloodstream infections | Clinical | Hospital facility | Outcome |
|  | CRBSI rate ^29^ | Bloodstream infections | Clinical | Hospital facility | Outcome |
|  | CRBSI rate of multi resistant pathogens ^29^ | Bloodstream infections | Clinical | Hospital facility | Outcome |
|  | CRBSI rate in hemato-oncology units ^29^ | Bloodstream infections | Clinical | Hospital facility | Outcome |
|  | CRBSI rate in non-ICU settings ^29^ | Bloodstream infections | Clinical | Hospital facility | Outcome |
|  | The percent of patients with severe sepsis or septic shock AND an unidentified organism who received vancomycin within 24 hrs following severe sepsis/ septic shock identification ^30^ | Bloodstream infections | Clinical | Hospital facility | Antibiotic prescribing/dispensing |
|  | Median time to initiation of vancomycin following severe sepsis/septic shock identification ^30^ | Bloodstream infections | Clinical | Hospital facility | Antibiotic prescribing/dispensing |
|  | The percent of patients with severe sepsis or septic shock AND an unidentified organism who received a recommended broad-spectrum antibiotic within 24 hrs following severe sepsis/septic shock identification ^30^ | Bloodstream infections | Clinical | Hospital facility | Antibiotic prescribing/dispensing |
|  | Median time to initiation of a recommended broad-spectrum antibiotic following severe sepsis/septic shock identification ^30^ | Bloodstream infections | Clinical | Hospital facility | Antibiotic prescribing/dispensing |
|  | The percent of patients with severe sepsis or septic shock AND an organism other than MRSA or MRSE who had vancomycin discontinued within 96 hrs following severe sepsis/septic shock identification ^30^ | Bloodstream infections | Clinical | Hospital facility | Antibiotic prescribing/dispensing |
|  | The percentage of patients with sepsis and an organism other than MRSA or MRSE (metacillin-resistant staphylococcus epidermis) who had vancomycin discontinued within 96 hours of diagnosis ^30^ | Bloodstream infections | Clinical | Hospital facility | Antibiotic prescribing/dispensing |
|  | Antibiotics prescribed by an ED provider for an admitted patient should be initiated while the patient is in the ED, in patients with sepsis or septic shock, administration of antibiotics should be initiated promptly in the ED aiming to reduce that time to as short a duration as feasible ^31^ | Bloodstream infections | Clinical | Hospital facility | Antibiotic prescribing/dispensing |
|  | Initial therapy should be intravenous (flu)cloxacillin (or nafcillin or oxacillin) or cefazolin in the case of methicillin-susceptible strains in patients with SAB ^32^ | Bloodstream infections | Clinical | Hospital facility | Antibiotic prescribing/dispensing |
|  | Hospital-wide incidence figures for nosocomial sepsis/bacteraemia available at least once per year ^23^ | Bloodstream infections | Clinical | Hospital facility | Outcome |
|  | Patients with severe sepsis/septic shock treated with antimicrobials in the first three hours. Formula: Number of patients with severe sepsis/septic shock, treated with antimicrobials in the first 3 hours / Total number of patients with severe sepsis/septic shock ^33^ | Bloodstream infections | Clinical | Hospital facility | Antibiotic prescribing/dispensing |
|  | Proportion of newborn babies who start antibiotic treatment for possible early-onset neonatal infection who have their need for it reassessed at 36 hours ^19^ | Bloodstream infections | Clinical | Hospital facility | Antibiotic prescribing/dispensing |
|  | Intravenous-to-oral switch should not be performed in uncomplicated SAB after 48–72 h ^32^ | Bloodstream infections | Clinical | Hospital facility | Antibiotic prescribing/dispensing |
|  | Intravenous-to-oral switch should not be performed in complicated SAB after 48–72 h ^32^ | Bloodstream infections | Clinical | Hospital facility | Antibiotic prescribing/dispensing |
|  | Rates of early-onset neonatal infection ^19^ | Bloodstream infections | Clinical | Hospital facility | Outcome |
|  | Appropriate duration of intravenous antibiotic treatment should be at least 14 days for uncomplicated SAB ^32^ | Bloodstream infections | Organisational | Hospital facility | Antibiotic prescribing/dispensing |
|  | Appropriate duration of intravenous antibiotic treatment should be at least 28 days for SAB complicated by metastatic abscesses or deep foci of infection ^32^ | Bloodstream infections | Organisational | Hospital facility | Antibiotic prescribing/dispensing |
|  | SAB should be documented in the medical discharge summary ^32^ | Bloodstream infections | Organisational | Hospital facility | Management |
|  | Collection of repeat blood cultures should be performed until first negative blood culture ^32^ | Bloodstream infections | Clinical | Hospital facility | Diagnostic process |
|  | Collection of follow-up blood cultures 4-7 days after collection of first blood culture that became positive (etiology of bacteremia/sepsis: Staphylococcus aureus or patients with fungemia) ^23^ | Bloodstream infections | Clinical | Hospital facility | Diagnostic process |
|  | Antibiotic therapy should be initiated within 24 h after first positive blood culture ^32^ | Bloodstream infections | Clinical | Hospital facility | Antibiotic prescribing/dispensing |
|  | Bacterial skin infections. - When presenting with multiple lesions: local Fucidin crème - When reduced resistance, fever and other general signs or worsening of the symptoms despite local treatment: oral flucloxacillin ^17^ | Skin infections | Clinical | Hospital facility, Primary health care | Antibiotic prescribing/dispensing |
|  | Proportion of patients presenting with impetigo that should be prescribed oral antibiotics ^7^ | Skin infections | Clinical | Primary health care | Antibiotic prescribing/dispensing |
|  | Bacterial skin infections. Erysipelas: first choice feniticillin, phenoxymethylpenicillin ^15^ | Skin infections | Clinical | Primary health care | Antibiotic prescribing/dispensing |
|  | Usage of systemic antibiotics in teeth extractions ^34^ | Dental infections | Clinical | Primary health care | Consumption of antibiotics/Prescription rate |
|  | Usage of systemic antibiotics in root canal treatments ^34^ | Dental infections | Clinical | Primary health care | Consumption of antibiotics/Prescription rate |
|  | Usage of systemic antibiotics in sharp tooth edges ^34^ | Dental infections | Clinical | Primary health care | Consumption of antibiotics/Prescription rate |
|  | Usage of systemic antibiotics in measures to preserve tooth vitality ^34^ | Dental infections | Clinical | Primary health care | Consumption of antibiotics/Prescription rate |
|  | Usage of systemic antibiotics in minor surgical interventions ^34^ | Dental infections | Clinical | Primary health care | Consumption of antibiotics/Prescription rate |
|  | Usage of systemic antibiotics when measuring PSI ^34^ | Dental infections | Clinical | Primary health care | Consumption of antibiotics/Prescription rate |
|  | Usage of systemic antibiotics in Local medical treatment ^34^ | Dental infections | Clinical | Primary health care | Consumption of antibiotics/Prescription rate |
|  | Usage of systemic antibiotics in fillings ^34^ | Dental infections | Clinical | Primary health care | Consumption of antibiotics/Prescription rate |
|  | Antibiotic usage in periodontal therapy ^34^ | Dental infections | Clinical | Primary health care | Consumption of antibiotics/Prescription rate |
|  | Antibiotic usage in dental care ^34^ | Dental infections | Clinical | Primary health care | Consumption of antibiotics/Prescription rate |
|  | Percentage of penicillin prescriptions in dental treatments ^34^ | Dental infections | Clinical | Primary health care | Consumption of antibiotics/Prescription rate |
|  | Percentage of clindamycin prescriptions in dental treatments ^34^ | Dental infections | Clinical | Primary health care | Consumption of antibiotics/Prescription rate |
|  | Percentage of tetracycline prescriptions in dental treatments ^34^ | Dental infections | Clinical | Primary health care | Consumption of antibiotics/Prescription rate |
|  | Usage of systemic antibiotics in dental treatments without indication for antibiotics ^34^ | Dental infections | Clinical | Primary health care | Antibiotic prescribing/dispensing |
|  | Proportion of patients (aged >2) that should be prescribed oral antibiotics when presenting with gastroenteritis ^7^ | Gastrointestinal infections | Clinical | Primary health care | Antibiotic prescribing/dispensing |
|  | The proportion of patients were prescribed prolonged antimicrobials following a surgery or procedure that is discordant with the current *Therapeutic Guidelines* or evidence-based, locally endorsed guidelines ^35^ | Surgical prophylaxis | Clinical | Hospital facility | Antibiotic prescribing/dispensing |
|  | Number of patients receiving surgical antibiotic prophylaxis according to guidelines / Total number of surgical patients receiving antibiotic prophylaxis ^3^ | Surgical prophylaxis | Clinical | Hospital facility | Antibiotic prescribing/dispensing |
|  | Surgeries with prophylaxis administered within 60 minutes prior to surgery / Total number of surgeries that require prophylaxis ^3^ | Surgical prophylaxis | Clinical | Hospital facility | Antibiotic prescribing/dispensing |
|  | Surgeries with prophylaxis stopped within 24 hours after surgery / Total number of surgeries that require prophylaxis ^3^ | Surgical prophylaxis | Clinical | Hospital facility | Antibiotic prescribing/dispensing |
|  | Percentage of single dose surgical antibiotic prophylaxis prescriptions that meet the NICE NG125 guidance regarding the choice of antibiotic for patients who have undergone elective colorectal surgery; by quarter ^2^ | Surgical prophylaxis | Clinical | Hospital facility | Antibiotic prescribing/dispensing |
|  | Percentage of surgical patients aged 18 years and older undergoing procedures with the indications for a first- OR second-generation cephalosporin prophylactic antibiotic who had an order for a first- OR second-generation cephalosporin for antimicrobial prophylaxis ^5^ | Surgical prophylaxis | Clinical | Hospital facility | Antibiotic prescribing/dispensing |
|  | Duration of surgical prophylaxis ^36^ | Surgical prophylaxis | Clinical | Hospital facility | Antibiotic prescribing/dispensing |
|  | Cumulative incidence of surgical interventions with postoperative surgical site infection ^4^ | Surgical prophylaxis | Clinical | Hospital facility | Outcome |
|  | The extent of prolonged prophylaxis (> 1 day), to prevent surgical site infections (SSIs) ^37^ | Surgical prophylaxis | Clinical | Hospital facility | Antibiotic prescribing/dispensing |
|  | Number of days of antimicrobials for surgical prophylaxis.  Formula: Number of days of use of antimicrobials for surgical prophylaxis / Total number of patients with surgical prophylaxis treatment × 100 ^33^ | Surgical prophylaxis | Clinical | Hospital facility | Antibiotic prescribing/dispensing |
|  | Surgical prophylaxis administered within the 60 minutes prior to surgery x 100 / Total number of surgeries requiring prophylaxis ^33^ | Surgical prophylaxis | Clinical | Hospital facility | Antibiotic prescribing/dispensing |
|  | Does your facility review surgical antimicrobial prophylaxis? If YES, are antimicrobial prescriptions for surgical antimicrobial prophylaxis compliant with facility-specific guideline in >95% >80% of sampled cases in your facility? ^26^ | Surgical prophylaxis | Organisational | Hospital facility | Antibiotic prescribing/dispensing |
|  | Surgical prophylaxis antibiotic therapy should be prescribed according to guideline ^38^ | Surgical prophylaxis | Organisational | Hospital facility | Antibiotic prescribing/dispensing |
|  | The availability of local guidelines (STGs) for surgical prophylaxis and adherence to these ^37^ | Surgical prophylaxis | Organisational | Hospital facility | Management |
|  | Prophylactic antibiotics should be added to a pre-operative checklist ^39^ | Surgical prophylaxis | Organisational | Hospital facility | Management |
|  | Proportion of surgical procedures for which antibiotic prophylaxis is indicated for which the person having surgery receives antibiotic prophylaxis in accordance with the local antibiotic formulary and that this is recorded ^19^ | Surgical prophylaxis | Organisational | Hospital facility | Antibiotic prescribing/dispensing |
|  | Surgical prophylaxis antibiotic therapy should be initiated within 1 h before incision ^38^ | Surgical prophylaxis | Organisational | Hospital facility | Antibiotic prescribing/dispensing |
|  | Antimicrobial prescribing rates ^19^ | NA | Clinical | General | Consumption of antibiotics/Prescription rate |
|  | Antimicrobial prescribing rates in hospitals ^19^ | NA | Clinical | Hospital facility | Consumption of antibiotics/Prescription rate |
|  | Antimicrobial prescribing rates in primary care ^19^ | NA | Clinical | Primary health care | Consumption of antibiotics/Prescription rate |
|  | Antibiotic prescribing rates (primary and secondary care) ^19^ | NA | Clinical | General | Consumption of antibiotics/Prescription rate |
|  | Total number of antibiotic prescriptions ^12^ | NA | Clinical | General | Consumption of antibiotics/Prescription rate |
|  | Consumption of antibacterials for systemic use expressed in DID (Defined daily doses per 1000 inhabitants per day) ^40^ | NA | Clinical | General | Consumption of antibiotics/Prescription rate |
|  | Total antibacterial use ^41^ | NA | Clinical | General | Consumption of antibiotics/Prescription rate |
|  | Total antibiotic use per 1000 persons per day ^42^ | NA | Clinical | General | Consumption of antibiotics/Prescription rate |
|  | Overall volume of antibiotics for systemic use prescribed (DDD) ^43^ | NA | Clinical | General | Consumption of antibiotics/Prescription rate |
|  | Treatments/courses per defined population ^44^ | NA | Clinical | General | Consumption of antibiotics/Prescription rate |
|  | Prescriptions per defined population ^28^ | NA | Clinical | General | Consumption of antibiotics/Prescription rate |
|  | DDDs per defined population ^28^ | NA | Clinical | General | Consumption of antibiotics/Prescription rate |
|  | Treatments/courses per defined number of physician contacts ^28^ | NA | Clinical | General | Consumption of antibiotics/Prescription rate |
|  | Prescriptions per defined number of physician contacts ^28^ | NA | Clinical | General | Consumption of antibiotics/Prescription rate |
|  | Percentage of all primary care consultations in which an antibiotic is prescribed or dispensed ^1^ | NA | Clinical | Primary health care | Consumption of antibiotics/Prescription rate |
|  | Seasonal variation of the total antibiotic consumption ^28^ | NA | Clinical | General | Consumption of antibiotics/Prescription rate |
|  | Defined daily doses (DDDs) per admission ^45^ | NA | Clinical | General | Consumption of antibiotics/Prescription rate |
|  | Days of therapy (DOT) per admission ^29^ | NA | Clinical | General | Consumption of antibiotics/Prescription rate |
|  | DDD per 100(0) patient-days ^3^ | NA | Clinical | General | Consumption of antibiotics/Prescription rate |
|  | DOTs per 1000 patient-days ^3^ | NA | Clinical | General | Consumption of antibiotics/Prescription rate |
|  | Total days of treatment for a specific disease / total number of cases treated ^9^ | NA | Clinical | General | Consumption of antibiotics/Prescription rate |
|  | Defined daily doses (DDDs) per (100 bed-days per CMIb) b CMI, case mix index.  This is a relative value assigned to a diagnosis-related group of patients in a medical care environment ^45^ | NA | Clinical | General | Consumption of antibiotics/Prescription rate |
|  | Prescribed daily doses (PDDs) per 100 PDs ^45^ | NA | Clinical | General | Consumption of antibiotics/Prescription rate |
|  | Total number of prescribed antibiotic items per STAR-PU by quarter ^2^ | NA | Clinical | General | Consumption of antibiotics/Prescription rate |
|  | Twelve-month rolling total number of prescribed antibiotic items per 1000 resident individuals per day ^2^ | NA | Clinical | General | Consumption of antibiotics/Prescription rate |
|  | Total number of prescribed antibiotic items per STAR-PU by quarter ^2^ | NA | Clinical | General | Consumption of antibiotics/Prescription rate |
|  | Twelve-month rolling total number of prescribed antibiotic items per STAR-PU ^2^ | NA | Clinical | General | Consumption of antibiotics/Prescription rate |
|  | Total number of prescribed antibiotic items per 1000 resident individuals by quarter ^45^ | NA | Clinical | General | Consumption of antibiotics/Prescription rate |
|  | Patients exposed to antibiotics per all patients ^45^ | NA | Clinical | General | Consumption of antibiotics/Prescription rate |
|  | Patients exposed to antibiotics per admission ^45^ | NA | Clinical | General | Consumption of antibiotics/Prescription rate |
|  | Average number of antibiotic prescriptions per member per year (PMPY) ^12^ | NA | Clinical | General | Consumption of antibiotics/Prescription rate |
|  | Average days supplied per antibiotic prescription ^12^ | NA | Clinical | General | Consumption of antibiotics/Prescription rate |
|  | Total number of prescriptions for antibiotics of concern ^12^ | NA | Clinical | General | Consumption of antibiotics/Prescription rate |
|  | Average number of prescriptions PMPY for antibiotics of concern ^12^ | NA | Clinical | General | Consumption of antibiotics/Prescription rate |
|  | Percentage of antibiotics of concern for all antibiotic prescriptions ^12^ | NA | Clinical | General | Consumption of antibiotics/Prescription rate |
|  | Average number of antibiotics PMPY reported by drug class for selected “antibiotics of concern” ^12^ | NA | Clinical | General | Consumption of antibiotics/Prescription rate |
|  | Average number of antibiotics PMPY reported by drug class for all other antibiotics ^12^ | NA | Clinical | General | Consumption of antibiotics/Prescription rate |
|  | Antimicrobial drug use data (as DDD/RDD or PDD per 100 occupied bed [patient] days and/or admission) available for several clinical divisions or departments (division-specific or aggregated for surgical and nonsurgical services and/or for general wards vs intensive care units) at least annually (in total and detailing the most important antibiotic classes) ^23^ | NA | Clinical | Hospital facility | Consumption of antibiotics/Prescription rate |
|  | Rate of oral vs. parenteral dispensed or prescribed daily doses of the most important and relevant drugs or drug classes available at least once per year for several clinical services ^23^ | NA | Clinical | General | Consumption of antibiotics/Prescription rate |
|  | Percentage of consumption IV versus IV + oral ^4^ | NA | Clinical | General | Consumption of antibiotics/Prescription rate |
|  | Antimicrobial use in the intensive care unit. Formula: Total number of days of use of antimicrobial agent /Total number of days of ICU patients ×100 ^33^ | NA | Clinical | Hospital facility | Consumption of antibiotics/Prescription rate |
|  | Days without antimicrobial use in ICU. Formula: Total number of ICU days without antimicrobials / Total number of days of ICU patients × 100 ^31^ | NA | Clinical | Hospital facility | Consumption of antibiotics/Prescription rate |
|  | Days free of antimicrobials in patients on antimicrobial treatment. Formula: Number of days free of antimicrobials in patients on antimicrobial treatment / Total days in ICU of patients on antimicrobial treatment × 100 ^31^ | NA | Clinical | Hospital facility | Consumption of antibiotics/Prescription rate |
|  | Volume of second line antibiotics as a share of total volume ^43^ | NA | Clinical | General | Consumption of antibiotics/Prescription rate |
|  | Recommended antibacterials ^41^ | NA | Clinical | General | Consumption of antibiotics/Prescription rate |
|  | Inappropriate DDDs / DDDs consumed ^9^ | NA | Clinical | General | Consumption of antibiotics/Prescription rate |
|  | Percentage of use of any antibiotic in the hospital and in the different wards ^46^ | NA | Clinical | Hospital facility | Consumption of antibiotics/Prescription rate |
|  | Percentage of use of the various types of antibiotics in the hospital and in various wards ^32^ | NA | Clinical | Hospital facility | Consumption of antibiotics/Prescription rate |
|  | The number of antibiotics prescribed for each patient ^46^ | NA | Clinical | General | Consumption of antibiotics/Prescription rate |
|  | Some antibiotics should be rarely prescribed ^11^ | NA | Clinical | General | Consumption of antibiotics/Prescription rate |
|  | Length of therapy (LOT) per admission ^45^ | NA | Clinical | Hospital facility | Consumption of antibiotics/Prescription rate |
|  | Length of therapy (LOT) per patient ^45^ | NA | Clinical | General | Consumption of antibiotics/Prescription rate |
|  | Frequency of use ^41^ | NA | Clinical | General | Consumption of antibiotics/Prescription rate |
|  | Prescribing by age ^25^ | NA | Clinical | General | Consumption of antibiotics/Prescription rate |
|  | Standardized antimicrobial administration ratio (SAAR) ^47^ | NA | Clinical | General | Consumption of antibiotics/Prescription rate |
|  | Proportion of people issued a back‑up (delayed) prescription for antimicrobials who are advised when to use the prescription ^19^ | NA | Clinical | General | Consumption of antibiotics/Prescription rate |
|  | Prescriptions of combination antibiotic therapies, expressed as a percentage of the total prescriptions of antibacterials for systemic use ^48^ | NA | Clinical | General | Consumption of antibiotics/Prescription rate |
|  | Total antibiotics DDD and cost in relation to standardized patients and time worked by GPs in the study period ^49^ | NA | Clinical | Primary health care | Consumption of antibiotics/Prescription rate |
|  | Relative first-line antibiotics prescription versus total antibiotics ^35^ | NA | Clinical | General | Consumption of antibiotics/Prescription rate |
|  | Consumption of cephalosporins expressed in DID ^40^ | NA | Clinical | General | Consumption of antibiotics/Prescription rate |
|  | Consumption of third and fourth generation of cephalosporines expressed as percentage ^40^ | NA | Clinical | General | Consumption of antibiotics/Prescription rate |
|  | Number of patients treated with cephalosporins ^14^ | NA | Clinical | General | Consumption of antibiotics/Prescription rate |
|  | Consumption of quinolones expressed in DID ^40^ | NA | Clinical | General | Consumption of antibiotics/Prescription rate |
|  | Selective use of fluoroquinolones (only as oral or in beta-lactam allergy/anaphylaxis) ^24^ | NA | Clinical | General | Consumption of antibiotics/Prescription rate |
|  | Ciprofloxacin use ^41^ | NA | Clinical | General | Consumption of antibiotics/Prescription rate |
|  | Seasonal variation of quinolone consumption ^40^ | NA | Clinical | General | Consumption of antibiotics/Prescription rate |
|  | Prescriptions of moxifloxacin expressed as a percentage of the total prescriptions of antibacterials for systemic use ^48^ | NA | Clinical | General | Consumption of antibiotics/Prescription rate |
|  | Prescriptions of levofloxacin, ofloxacin or ciprofloxacin among patients having been prescribed a quinolone (J01M) in the preceding 6 months, expressed as a percentage of the total prescriptions of quinolones ^34^ | NA | Clinical | General | Consumption of antibiotics/Prescription rate |
|  | Prescriptions of first-generation quinolones, expressed as a percentage of the total prescriptions  of antibacterials for systemic use ^48^ | NA | Clinical | General | Consumption of antibiotics/Prescription rate |
|  | Prescriptions of moxifloxacin or levofloxacin associated on the same day with another antibiotic, expressed as a percentage  of the total prescriptions of antibacterials for systemic use ^48^ | NA | Clinical | General | Consumption of antibiotics/Prescription rate |
|  | Prescriptions of nitrofurantoine (J01XE01), first-generation quinolones or fosfomycin–trometamol prescriptions in male patients, expressed as a percentage of the total prescriptions of antibacterials for systemic use in male patients ^48^ | NA | Clinical | General | Consumption of antibiotics/Prescription rate |
|  | Consumption of penicillins expressed in DID ^40^ | NA | Clinical | General | Consumption of antibiotics/Prescription rate |
|  | Consumption of b-lactamase sensitive penicillins expressed as percentage ^40^ | NA | Clinical | General | Consumption of antibiotics/Prescription rate |
|  | Amoxicillin use ^41^ | NA | Clinical | General | Consumption of antibiotics/Prescription rate |
|  | Flucloxacillin use ^25^ | NA | Clinical | General | Consumption of antibiotics/Prescription rate |
|  | Phenoxymethylpenicillin use ^25^ | NA | Clinical | General | Consumption of antibiotics/Prescription rate |
|  | Co-amoxiclav use ^25^ | NA | Clinical | General | Consumption of antibiotics/Prescription rate |
|  | Consumption of combination of penicillins, including b-lactamase inhibitor expressed as percentage ^40^ | NA | Clinical | General | Consumption of antibiotics/Prescription rate |
|  | Proportion of amoxicillin users (amoxicillin index) ^50^ | NA | Clinical | General | Consumption of antibiotics/Prescription rate |
|  | Relative number of amoxycillin prescriptions versus amoxycillin/clavulanate prescriptions ^49^ | NA | Clinical | General | Consumption of antibiotics/Prescription rate |
|  | Number of patients treated with narrow-spectrum penicillin ^14^ | NA | Clinical | General | Consumption of antibiotics/Prescription rate |
|  | Percentage of prescribed antibiotic items from cephalosporin, quinolone and co-amoxiclav class by quarter ^2^ | NA | Clinical | General | Consumption of antibiotics/Prescription rate |
|  | Twelve-month rolling percentage of prescribed antibiotic items from cephalosporin, quinolone and co-amoxiclav class ^2^ | NA | Clinical | General | Consumption of antibiotics/Prescription rate |
|  | Number of patients treated with broad-spectrum penicillin + /– clavulanic acid ^14^ | NA | Clinical | General | Consumption of antibiotics/Prescription rate |
|  | Ratio between broad-spectrum beta-lactam versus non-broad-spectrum beta-lactams per discipline ^4^ | NA | Clinical | General | Consumption of antibiotics/Prescription rate |
|  | Ratio between users of amoxicillin to broad-spectrum penicillins, cephalosporins and macrolides (A/B ratio) ^50^ | NA | Clinical | General | Consumption of antibiotics/Prescription rate |
|  | Ratio of the consumption of broad to the consumption of narrow spectrum penicillins, cephalosporins and macrolide ^40^ | NA | Clinical | General | Consumption of antibiotics/Prescription rate |
|  | Percentage of broad-spectrum prescribed antibiotic items (cephalosporin, quinolone and co-amoxiclav class) by quarter ^2^ | NA | Clinical | General | Consumption of antibiotics/Prescription rate |
|  | Proportion of cephalosporins and quinolones, of total antibiotic drugs used ^42^ | NA | Clinical | General | Consumption of antibiotics/Prescription rate |
|  | Volume of cephalosporines and quinolones as a proportion of all systemic antibiotics prescribed (DDD) ^43^ | NA | Clinical | General | Consumption of antibiotics/Prescription rate |
|  | Consumption of macrolides, lincosamides and streptogramins expressed in DID ^40^ | NA | Clinical | General | Consumption of antibiotics/Prescription rate |
|  | Total tetracycline use ^41^ | NA | Clinical | General | Consumption of antibiotics/Prescription rate |
|  | Doxycycline use ^41^ | NA | Clinical | General | Consumption of antibiotics/Prescription rate |
|  | Total macrolide use ^2^ | NA | Clinical | General | Consumption of antibiotics/Prescription rate |
|  | Percentage of erythromycin among total macrolides prescribed ^49^ | NA | Clinical | General | Consumption of antibiotics/Prescription rate |
|  | Number of patients treated with macrolides ^14^ | NA | Clinical | General | Consumption of antibiotics/Prescription rate |
|  | Ratio of co-trimoxazole items to trimethoprim items ^41^ | NA | Clinical | General | Consumption of antibiotics/Prescription rate |
|  | Trimethoprim use ^41^ | NA | Clinical | General | Consumption of antibiotics/Prescription rate |
|  | Co-trimoxazole use ^41^ | NA | Clinical | General | Consumption of antibiotics/Prescription rate |
|  | Twelve-month rolling proportion of trimethoprim class prescribed antibiotic items as a ratio of trimethoprim to nitrofurantoin ^2^ | NA | Clinical | General | Consumption of antibiotics/Prescription rate |
|  | Nitrofurantoin use ^41^ | NA | Clinical | General | Consumption of antibiotics/Prescription rate |
|  | Carbapenem prescribing DDDs per 1000 admissions by quarter and acute trust ^2^ | NA | Clinical | General | Consumption of antibiotics/Prescription rate |
|  | % of prescriptions with an antibiotic ^51^ | NA | Clinical | General | Consumption of antibiotics/Prescription rate |
|  | Clindamycin use ^2^ | NA | Clinical | General | Consumption of antibiotics/Prescription rate |
|  | Prevalence of blood cultures in a hospital ^29^ | NA | Clinical | Hospital facility | Diagnostic process |
|  | Clostridium difficile toxin test available within 18 h ^4^ | NA | Clinical | General | Diagnostic process |
|  | Legionella urinary antigen test available within 18 h ^4^ | NA | Clinical | General | Diagnostic process |
|  | The results of bacteriological sensitivity(s) is documented ^52^ | NA | Clinical | General | Management |
|  | Obtain sputum samples for Gram stain and culture ^21^ | NA | Clinical | General | Diagnostic process |
|  | Performance of urine culture ^24^ | NA | Clinical | General | Diagnostic process |
|  | Perform surveillance cultures if selective digestive or oropharyngeal decontamination is applied at the ICU ^53^ | NA | Clinical | Hospital facility | Diagnostic process |
|  | Follow-up blood cultures after initiation of antimicrobial therapy should be done regardless of clinical evolution ^32^ | NA | Clinical | Hospital facility | Diagnostic process |
|  | Number of patients with an indication receiving empirical treatment with antibiotic(s) according to clinical guidelines / Total number of patients with this indication ^3^ | NA | Clinical | General | Antibiotic prescribing/dispensing |
|  | Antibiotic prescriptions that deviate from guidelines should be justified ^39^ | NA | Clinical | General | Antibiotic prescribing/dispensing |
|  | Initial therapy (drugs) according to local / national guideline ^23^ | NA | Clinical | General | Antibiotic prescribing/dispensing |
|  | Indications for a disease based on clinical guidelines x 100 / total number of indications for that disease ^9^ | NA | Clinical | General | Antibiotic prescribing/dispensing |
|  | The proportion of prescriptions for restricted antimicrobials that are in accordance with the locally endorsed approval policy ^35^ | NA | Clinical | General | Antibiotic prescribing/dispensing |
|  | Number of deaths by type of infection / Total number of patients with that infection ^9^ | NA | Clinical | General | Outcome |
|  | Days of hospitalization by type of infection / Total number of patients with that infection ^3,9^ | NA | Clinical | Hospital facility | Outcome |
|  | Possible contraindications should be taken into account when antibiotics are prescribed ^39^ | NA | Clinical | General | Antibiotic prescribing/dispensing |
|  | Proportion of newborn babies who need antibiotic treatment who receive it within 1 hour of the decision to treat ^19^ | NA | Clinical | Hospital facility | Antibiotic prescribing/dispensing |
|  | Whether antimicrobial prescription was empiric or targeted based on an identified pathogen ^36^ | NA | Clinical | General | Antibiotic prescribing/dispensing |
|  | Timely initiation of antibiotic therapy within four hours after presentation ^21^ | NA | Clinical | General | Antibiotic prescribing/dispensing |
|  | Initiation of treatment within 4 hours after clinical presentation ^24^ | NA | Clinical | General | Antibiotic prescribing/dispensing |
|  | A selective susceptibly report (or antibiogram) is a report of a selection of antibiotic sensitivities, based on bacteriological activity, broadness of spectrum or toxicity ^39^ | NA | Clinical | General | Management |
|  | The prescribed antibiotic should be active against all the likely causative pathogens ^39^ | NA | Clinical | General | Antibiotic prescribing/dispensing |
|  | Prevalence of empiric antibiotic use as depicted by the frequency of requesting for sensitivity analyses ^45^ | NA | Clinical | General | Antibiotic prescribing/dispensing |
|  | When prescribing antibiotics in the ED, the following should be taken into account: Relevant results of previous cultures and susceptibilities ^31^ | NA | Clinical | Hospital facility | Antibiotic prescribing/dispensing |
|  | When prescribing antibiotics in the ED, the following should be taken into account: Previous antibiotic use ^31^ | NA | Clinical | Hospital facility | Antibiotic prescribing/dispensing |
|  | When prescribing antibiotics in the ED, the following should be documented in the medical record: previous antibiotic use (including date and duration) ^31^ | NA | Clinical | Hospital facility | Antibiotic prescribing/dispensing |
|  | Number of patients where a 48-hour review is performed / Total number of patients treated with antibiotic(s) hospitalized >48 hours ^3^ | NA | Clinical | Hospital facility | Antibiotic prescribing/dispensing |
|  | Number of patients where a de-escalation from the initial therapy is performed / Total number of indicated empirical treatments ^3^ | NA | Clinical | General | Antibiotic prescribing/dispensing |
|  | Number of regimens switched to oral route / Total number of regimens that can be switched to oral route based on predefined criteria ^3^ | NA | Clinical | General | Antibiotic prescribing/dispensing |
|  | Prompt switching of intravenous (IV) antimicrobial treatment to the oral route of administration as soon as patients meet switch criteria ^18^ | NA | Clinical | General | Antibiotic prescribing/dispensing |
|  | Altered or withdrawn prescriptions for antimicrobials following microbiological results showing lack of effectiveness of initial antimicrobial treatment ^19^ | NA | Clinical | General | Antibiotic prescribing/dispensing |
|  | The choice of antimicrobial treatment is reviewed according to clinical response and/or sensitivities ^52^ | NA | Clinical | General | Antibiotic prescribing/dispensing |
|  | The intravenous route of administration is switched to the oral route at an appropriate time (48 h after initiation and if not clinically indicated, requirement for intravenous route is reviewed every 24 h) ^52^ | NA | Clinical | General | Antibiotic prescribing/dispensing |
|  | Antimicrobial treatment is discontinued on completion of the documented course ^52^ | NA | Clinical | General | Antibiotic prescribing/dispensing |
|  | If no IVOS within first 48 h, review daily thereafter ^28^ | NA | Clinical | General | Antibiotic prescribing/dispensing |
|  | Antibiotic therapy should be discontinued based on the lack of clinical evidence of infection ^39^ | NA | Clinical | General | Antibiotic prescribing/dispensing |
|  | Antibiotics for empirical therapy should be reviewed within the third day of treatment or when microbiological results become available ^39^ | NA | Clinical | General | Antibiotic prescribing/dispensing |
|  | Antibiotics should be continued in the ICU until assessed within 48 hours (before considering de-escalation) ^39^ | NA | Clinical | Hospital facility | Antibiotic prescribing/dispensing |
|  | Prevalence of empiric antibiotic use as depicted by the frequency of requesting for sensitivity analyses ^45^ | NA | Clinical | General | Antibiotic prescribing/dispensing |
|  | Discontinuation of specified antimicrobial prescriptions after a predefined duration? ^26^ | NA | Clinical | General | Antibiotic prescribing/dispensing |
|  | Was there a review of the diagnosis? ^54^ | NA | Clinical | General | Antibiotic prescribing/dispensing |
|  | If positive microbiological results were available, was there any adaptation of the antibiotic treatment, for example streamlining or discontinuation? ^54^ | NA | Clinical | General | Antibiotic prescribing/dispensing |
|  | The extent of empiric versus targeted antimicrobial treatment ^37^ | NA | Clinical | General | Antibiotic prescribing/dispensing |
|  | The extent of switching from IV to oral antibiotics ^37^ | NA | Clinical | General | Antibiotic prescribing/dispensing |
|  | Non-empirical antimicrobial use.  Formula: Total antimicrobials used to treat infections in a directed manner /Total of antimicrobials used to treat infections ×100 ^33^ | NA | Clinical | General | Consumption of antibiotics/Prescription rate |
|  | Changes in antimicrobials used as treatment. Formula: Total number of antimicrobials changed to another antimicrobial / Total of antimicrobials used to treat infections × 100 ^33^ | NA | Clinical | General | Antibiotic prescribing/dispensing |
|  | Empirical antimicrobials changed because they are inadequate. Formula: Number of empirical antimicrobials changed because they are inadequate Total number of empirical antimicrobials used to treat infections × 100 ^33^ | NA | Clinical | General | Antibiotic prescribing/dispensing |
|  | Empirical antimicrobial changed for de-escalation.  Formula: Number of empirical antimicrobials changed by adjustment or de-escalation Total number of empirical antimicrobials used to treat infections × 100 ^33^ | NA | Clinical | General | Antibiotic prescribing/dispensing |
|  | Number of de-escalations from empiric therapy x 100 / Total number of indicated empiric treatments ^9^ | NA | Clinical | General | Antibiotic prescribing/dispensing |
|  | Number of effectively switched regimens to oral route x 100 / Total number of regimens that can be switched to oral route ^9^ | NA | Clinical | General | Antibiotic prescribing/dispensing |
|  | Number of patients with a written indication for antibiotic treatment / Total number of patients treated with antibiotic(s) ^3^ | NA | Clinical | General | Management |
|  | Number of patients with a written stop/review date for antibiotic treatment / Total number of patients treated with antibiotic(s) ^3^ | NA | Clinical | General | Management |
|  | The indication for prescribing an oral antimicrobial as a result of an intravenous to oral switch is documented in the case notes or the inpatient medication administration chart ^52^ | NA | Clinical | General | Management |
|  | The intended duration of oral antimicrobial treatment as a result of an intravenous to oral switch is documented ^52^ | NA | Clinical | General | Management |
|  | Identified interactions between antimicrobial regimen and concurrent medications are documented with a recommended management plan of the interaction ^52^ | NA | Clinical | General | Management |
|  | The intended duration of antimicrobial treatment is documented ^52^ | NA | Clinical | General | Management |
|  | Percentage of patients with reason for antibiotic use in notes and stop/review date documented ^55^ | NA | Clinical | General | Management |
|  | Recording of the rationale for the initial antimicrobial prescription and any subsequent changes ^37^ | NA | Clinical | General | Management |
|  | The proportion of patients with an adverse reaction to an antimicrobial with comprehensive documentation of the reaction in their healthcare record ^35^ | NA | Clinical | General | Management |
|  | The proportion of prescriptions for which an antimicrobial review and updated treatment decision is documented within 48 hours from the first prescription ^35^ | NA | Clinical | General | Antibiotic prescribing/dispensing |
|  | Items/STAR-PU for antibiotics ^56^ | NA | Clinical | General | Antibiotic prescribing/dispensing |
|  | Prescribed antibiotics should be chosen from an essential list/formulary ^9^ | NA | Clinical | General | Antibiotic prescribing/dispensing |
|  | % of drugs prescribed from essential drug list or formulary ^51^ | NA | Clinical | General | Consumption of antibiotics/Prescription rate |
|  | Oral antimicrobial therapy should always be used in preference to intravenous therapy where these have equivalent efficacy unless there are other relevant factors, e.g. toxicity, lack of oral route, allergies or drug–drug or drug–patient interactions ^57^ | NA | Clinical | General | Antibiotic prescribing/dispensing |
|  | Prescribed antibiotics should actually be administered to the patients ^39^ | NA | Clinical | General | Antibiotic prescribing/dispensing |
|  | Prescriptions concerning restricted / alert anti-infectives from a defined list are approved for specific patients, not generically ^23^ | NA | Clinical | General | Antibiotic prescribing/dispensing |
|  | Oral administration of drugs with high bioavailability (fluoroquinolones [exept norfloxacin], clindamycin, doxycycline, linezolid, metronidazole, rifampin, fluconazole, voriconazole) (not for patients with resorption disorders, short bowel syndrome, emesis, severe sepsis / septic shock) ^23^ | NA | Clinical | General | Antibiotic prescribing/dispensing |
|  | Inappropriate empirical antimicrobial treatment. Formula: Total number of inappropriate empirical antimicrobials / Total number of empirical antimicrobials used to treat infections × 100 ^33^ | NA | Clinical | General | Antibiotic prescribing/dispensing |
|  | Antibiotic generic prescribing rate (%) ^56^ | NA | Clinical | General | Consumption of antibiotics/Prescription rate |
|  | Days of hospitalization by type of infection / Total number of patients with that infection ^9^ | NA | Clinical | Hospital facility | Outcome |
|  | Antibacterials associated with a higher risk of Clostridium difficile infection – total use ^41^ | NA | Clinical | General | Consumption of antibiotics/Prescription rate |
|  | Number of health-care-associated C. difficile infections in a period of time / Total number of patient days within that period x 100 000 ^3^ | NA | Clinical | Hospital facility | Outcome |
|  | Rates of nosocomial Clostridium difficile should be monitored at the health care facility ^39^ | NA | Clinical | Hospital facility | Outcome |
|  | Incidence figures for *C. difficile* associated diarrhoea available for several clinical services (division-specific and/or general wards vs. intensive care units) at least once per year ^23^ | NA | Clinical | Hospital facility | Outcome |
|  | Twelve antibacterials (Amoxycillin, ampicillin, cephalexin, co-amoxiclav, erythromycin, flucloxacillin, metronidazole, nitrofurantoin, oxytetracycline, phenoxymethylpenicillin, tetracycline and trimethoprim) as a proportion of BNF section 5.1 drugs ^58^ | NA | Clinical | General | Consumption of antibiotics/Prescription rate |
|  | Empirical systemic antibiotic therapy should be prescribed according to the institutional, national, or international guideline ^38^ | NA | Organisational | Generic | Antibiotic prescribing/dispensing |
|  | Empirical antibiotic therapy should be changed to pathogen-directed therapy if culture results become available ^38^ | NA | Organisational | Generic | Antibiotic prescribing/dispensing |
|  | Compliance with guidelines for documented indication (where guidance was available) ^36^ | NA | Organisational | Generic | Antibiotic prescribing/dispensing |
|  | The route of administration of antimicrobial is compliant with local policy ^52^ | NA | Organisational | Generic | Antibiotic prescribing/dispensing |
|  | The dosage regimen of prescribed antimicrobial is compliant with local policy ^52^ | NA | Organisational | Generic | Antibiotic prescribing/dispensing |
|  | Antibiotics should be prescribed according to national guidelines when no local guidelines are available ^39^ | NA | Organisational | Generic | Antibiotic prescribing/dispensing |
|  | Antibiotic prescribing should be compliant with recommendations from infectious disease and/or microbiology specialist(s) ^39^ | NA | Organisational | Generic | Antibiotic prescribing/dispensing |
|  | Microbiological investigations should be performed according to guidelines ^39^ | NA | Organisational | Generic | Diagnostic process |
|  | Duration of antibiotic therapy should be compliant with guidelines ^39^ | NA | Organisational | Generic | Antibiotic prescribing/dispensing |
|  | Switching from intravenous to oral antibiotic(s) should be performed according to guidelines ^39^ | NA | Organisational | Generic | Antibiotic prescribing/dispensing |
|  | Percentage of guideline compliance (at each patient level) ^55^ | NA | Organisational | Generic | Antibiotic prescribing/dispensing |
|  | Prevalence of prescribing according to local guidelines where they exist ^45^ | NA | Organisational | Generic | Antibiotic prescribing/dispensing |
|  | Proportion of prescriptions for antimicrobials with the clinical indication, dose and duration of treatment documented ^19^ | NA | Organisational | Hospital facility, Primary health care | Management |
|  | Antibiotics that are dispensed to outpatients should be adequately labelled (patient name, antibiotics name, when antibiotics should be taken) ^10^ | NA | Organisational | Primary health care | Management |
|  | Adherence to ASP recommendations ^59^ | NA | Organisational | Generic | Antibiotic prescribing/dispensing |
|  | Indication for antimicrobial use documented in the patient notes ^36^ | NA | Organisational | Generic | Management |
|  | Outpatients should receive antibiotic therapy in compliance with guidelines; this includes, but is not limited to indication, choice of antibiotic, duration, dose and timing ^9^ | NA | Organisational | Primary health care | Antibiotic prescribing/dispensing |
|  | Stop or review date for antimicrobial use documented in the notes (and medicine chart) ^36^ | NA | Organisational | Generic | Management |
|  | Proportion of prescriptions for antimicrobials issued to people admitted to hospital with a record of a microbiological sample being taken ^19^ | NA | Organisational | Hospital facility | Diagnostic process |
|  | Proportion of prescriptions for antimicrobials issued to people admitted to hospital and reviewed when microbiological results become available ^19^ | NA | Organisational | Hospital facility | Antibiotic prescribing/dispensing |
|  | Diagnostic microbiological tests relevant for the site of infection should be collected in the ED preferably before antibiotic administration ^31^ | NA | Organisational | Hospital facility | Diagnostic process |
|  | An antibiotic plan should be documented in the medical record at the start of the antibiotic treatment.  Antibiotic plan includes: indication, name, doses, duration, route, and interval of administration. ^39^ | NA | Organisational | Generic | Management |
|  | Back-up (delayed) prescriptions for antimicrobials that are dispensed ^19^ | NA | Organisational | Generic | Antibiotic prescribing/dispensing |
|  | Annual analysis of AB consumption data (in DDD or RDD) available on hospital level by drug/drug class ^4^ | NA | Organisational | Hospital facility | Consumption of antibiotics/Prescription rate |
|  | Annual analysis of AB consumption data (in DDD or RDD) available on department level (i.e. by discipline) ^4^ | NA | Organisational | Hospital facility | Consumption of antibiotics/Prescription rate |
|  | Annual analysis of AB consumption data (in DDD or RDD) available on ward level ^4^ | NA | Organisational | Hospital facility | Consumption of antibiotics/Prescription rate |
|  | Drug use Total annual antibacterial (ATC J01) consumption for monitoring local temporal trend ^4^ | NA | Organisational | Generic | Consumption of antibiotics/Prescription rate |
|  | Does your facility monitor antimicrobial use by grams (Defined Daily Dose [DDD]) or counts (Days of Therapy [DOT]) of antimicrobial(s) by patients per days? ^26^ | NA | Organisational | Generic | Consumption of antibiotics/Prescription rate |
|  | Does your hospital monitor the quantity of antimicrobials prescribed / dispensed/purchased at the unit and/or hospital wide level? ^50^ | NA | Organisational | Hospital facility | Consumption of antibiotics/Prescription rate |
|  | Antibiotic use should be preferably expressed in at least two metrics simultaneously ^45^ | NA | Organisational | Generic | Consumption of antibiotics/Prescription rate |
|  | The results of bacteriological sensitivities should be documented in the medical records ^39^ | NA | Organisational | Hospital facility | Management |
|  | Antibiotics should not be sold without prescription ^11^ | NA | Organisational | Generic | Antibiotic prescribing/dispensing |
|  | Suitable oral switch option available, considering oral bioavailability, any clinically significant drug interactions or patient allergies ^28^ | NA | Organisational | Primary health care | Antibiotic prescribing/dispensing |
|  | Antibiotics prescribed by an ED provider for an admitted patient should be initiated while the patient is in the ED ^31^ | NA | Organisational | Hospital facility | Antibiotic prescribing/dispensing |
|  | Nurse prescribing ^41^ | NA | Workforce | General | Consumption of antibiotics/Prescription rate |
|  | Dental prescribing ^41^ | NA | Workforce | Primary health care | Consumption of antibiotics/Prescription rate |

ABRS = acute bacterial rhinosinusitis; BTS=British Thoracic Society; CAP = Community-acquired pneumonia; ED = Emergency Department; HAP = Hospital-acquired pneumonia NA = not applicable; PSI = Periodontal Screening Index; SAB = *Staphylococcus aureus* bacteraemia; STAR-PU=specific therapeutic groups age sex related prescribing unit; UTI = Urinary tract infection.

**Table S4**. Final set of non AWaRe-based quality indicators and quantity metrics included in our review.

| N | Indicator | Type of infection | Type | Setting | Target |
| --- | --- | --- | --- | --- | --- |
|  | Patients with acute bronchitis. Number of patients with known penicillin allergy treated with macrolides / Number of patients treated with macrolides ^6^ | RTIs | Clinical | Primary health care | Antibiotic prescribing/dispensing |
|  | Patients with acute pharyngotonsillitis. Number of patients with known penicillin allergy treated with macrolides / Number of patients treated with macrolides ^6^ | RTIs | Clinical | Primary health care | Antibiotic prescribing/dispensing |
|  | Patients with acute rhinosinusitis. Number of patients with known penicillin allergy treated with macrolides / Number of patients treated with macrolides ^6^ | RTIs | Clinical | Primary health care | Antibiotic prescribing/dispensing |
|  | Otitis externa with fever and other general signs: oral flucloxacillin ^17^ | RTIs | Clinical | Primary health care | Antibiotic prescribing/dispensing |
|  | Patients with acute otitis media. Number of patients with known penicillin allergy treated with macrolides / Number of patients treated with macrolides ^6^ | RTIs | Clinical | Primary health care | Antibiotic prescribing/dispensing |
|  | Percentage of patients aged 2 years and older with a diagnosis of AOE who were prescribed topical preparations ^5^ | RTIs | Clinical | Primary health care | Antibiotic prescribing/dispensing |
|  | Patients with pneumonia. Number of patients with known penicillin allergy treated with macrolides / Number of patients treated with macrolides ^6^ | RTIs | Clinical | Hospital facility, Primary health care | Antibiotic prescribing/dispensing |
|  | Evidence of local arrangements to ensure that adults with community‑acquired pneumonia who are admitted to hospital start antibiotic therapy within 4 hours of presentation ^19^ | RTIs | Organisational | Hospital facility | Management |
|  | Does your facility have treatment recommendations, based on national guidelines and local susceptibility, to assist with antimicrobial selection for CAP? ^26^ | RTIs | Organisational | Hospital facility, Primary health care | Management |
|  | Evidence of local prescribing protocols to direct antibiotic prescribing in children with bronchiolitis ^19^ | RTIs | Organisational | Hospital facility, Primary health care | Management |
|  | Relative prescribing of urinary antiseptics versus quinolones ^49^ | UTIs | Clinical | Hospital facility, Primary health care | Antibiotic prescribing/dispensing |
|  | Proportion of episodes of suspected UTI in adults with indwelling urinary catheters that are investigated using dipstick testing ^19^ | UTIs | Clinical | Hospital facility, Primary health care | Diagnostic process |
|  | Proportion of men with a recurrent UTI who are referred for specialist advice ^19^ | UTIs | Clinical | Primary health care | Diagnostic process |
|  | Proportion of women with a recurrent lower UTI where the underlying cause is unknown following clinical assessment who are referred for specialist advice ^19^ | UTIs | Clinical | Primary health care | Diagnostic process |
|  | Proportion of women with a recurrent upper UTI referred for specialist advice ^19^ | UTIs | Clinical | Hospital facility | Diagnostic process |
|  | Number of recurrent UTIs in adults ^19^ | UTIs | Clinical | Primary health care | Outcome |
|  | Proportion of infants, children and young people with a UTIs who have risk factors for urinary tract infection and serious underlying pathology recorded as part of their history and examination ^19^ | UTIs | Clinical | Hospital facility, Primary health care | Management |
|  | Proportion of infants, children and young people with a urinary tract infection caused by coliform bacteria who have results of microbiology laboratory testing differentiated by E. coli or non-E. coli organisms ^19^ | UTIs | Clinical | Hospital facility, Primary health care | Management |
|  | Proportion of children and young people who have had a urinary tract infection who receive information about how to recognise re-infection and to seek medical advice straight away ^19^ | UTIs | Clinical | Hospital facility, Primary health care | Management |
|  | Does your facility have treatment recommendations, based on national guidelines and local susceptibility, to assist with antimicrobial selection for UTIs? ^26^ | UTIs | Organisational | Hospital facility, Primary health care | Management |
|  | Evidence of a written protocol to ensure that people who need a urinary catheter have their risk of infection minimised by the completion of specified procedures necessary for the safe insertion and maintenance of the catheter and its removal as soon as it is no longer needed ^19^ | UTIs | Organisational | General | Management |
|  | Evidence of a register of people with an indwelling urinary catheter in the community ^19^ | UTIs | Organisational | General | Management |
|  | Evidence of local arrangements to ensure that infants, children and young people (under 16 years) with a urinary tract infection have risk factors for urinary tract infection and serious underlying pathology recorded as part of their history and examination ^19^ | UTIs | Organisational | General | Management |
|  | Evidence of local arrangements to ensure that children and young people (under 16 years) who have had a urinary tract infection are given information about how to recognise re-infection and to seek medical advice straight away ^19^ | UTIs | Organisational | General | Management |
|  | Do not prescribe antibiotic prophylaxis to patients with a urinary catheter in place ^24^ | UTIs | Organisational | General | Antibiotic prescribing/dispensing |
|  | Consider all diabetic patients with cystitis as having a complicated UTI and treat with empiric treatment according to national guidelines ^24^ | UTIs | Organisational | Hospital facility, Primary health care | Antibiotic prescribing/dispensing |
|  | Proportion of people with suspected sepsis in acute hospital settings who are seen by a consultant if their condition fails to respond within 1 hour of initial intravenous antibiotics or fluid bolus ^19^ | Bloodstream infections | Clinical | Hospital facility | Management |
|  | Evidence of local arrangements to ensure that a structured set of observations are used to stratify risk of severe illness or death from sepsis ^19^ | Bloodstream infections | Organisational | Hospital facility | Management |
|  | Evidence of local arrangements to ensure urgent assessment mechanisms are in place to deliver antibiotics to people with suspected sepsis in acute hospital settings within 1 hour of any high-risk criteria of severe illness or death from sepsis being identified ^19^ | Bloodstream infections | Organisational | Hospital facility | Management |
|  | Evidence of local arrangements and written clinical protocols to ensure that people with suspected sepsis in acute hospital settings have their lactate levels recorded ^19^ | Bloodstream infections | Organisational | Hospital facility | Management |
|  | Evidence of acute hospital settings having arrangements in place which ensure that people with suspected sepsis are seen by a consultant if their condition fails to respond within 1 hour of initial intravenous antibiotics or fluid bolus. This includes ensuring a consultant is available to attend promptly. ^19^ | Bloodstream infections | Organisational | Hospital facility | Management |
|  | Evidence of local arrangements to ensure that information about symptoms to monitor and how to access medical care if needed is available to people with suspected sepsis who have been stratified as being at low risk of severe illness or death ^19^ | Bloodstream infections | Organisational | Hospital facility | Management |
|  | Proportion of people with suspected sepsis who have been stratified as being at low risk of severe illness or death who are given information about symptoms to monitor and how to access medical care ^19^ | Bloodstream infections | Organisational | Hospital facility | Management |
|  | Evidence of local arrangements to ensure that pregnant women whose babies are at risk of early‑onset neonatal infection are offered intrapartum antibiotic prophylaxis and given the first dose as soon as possible ^19^ | Bloodstream infections | Organisational | Hospital facility | Management |
|  | Proportion of pregnant women whose babies are at risk of early-onset neonatal infection who receive intrapartum antibiotic prophylaxis ^19^ | Bloodstream infections | Organisational | Hospital facility | Antibiotic prescribing/dispensing |
|  | Proportion of pregnant women receiving intrapartum antibiotic prophylaxis who are given it as soon as possible ^19^ | Bloodstream infections | Organisational | Hospital facility | Antibiotic prescribing/dispensing |
|  | Evidence of local arrangements and written protocols to ensure that pregnant women and newborn babies receive a comprehensive clinical assessment for the risks or indicators of early-onset neonatal infection ^19^ | Bloodstream infections | Organisational | Hospital facility | Management |
|  | Proportion of pregnant women who are assessed for risk factors for early-onset neonatal infection ^19^ | Bloodstream infections | Organisational | Hospital facility | Management |
|  | Proportion of newborn babies who are assessed for clinical indicators of early-onset neonatal infection ^19^ | Bloodstream infections | Organisational | Hospital facility | Management |
|  | Proportion of newborn babies with risk factors or clinical indicators of early-onset neonatal infection who receive an immediate physical examination including an assessment of the vital signs ^19^ | Bloodstream infections | Organisational | Hospital facility | Management |
|  | Evidence of local arrangements to ensure that newborn babies who need antibiotic treatment receive it within 1 hour of the decision to treat ^19^ | Bloodstream infections | Organisational | Hospital facility | Management |
|  | Evidence of local arrangements to ensure that newborn babies who start antibiotic treatment for possible early-onset neonatal infection have their need for it reassessed at 36 hours ^19^ | Bloodstream infections | Organisational | Hospital facility | Management |
|  | Evidence of local arrangements and protocols to ensure that parents or carers of newborn babies in whom early-onset neonatal infection has been a concern are given verbal and written information about neonatal infection before discharge ^19^ | Bloodstream infections | Organisational | Hospital facility | Management |
|  | Proportion of parents or carers of newborn babies in whom early-onset neonatal infection has been a concern who are given verbal and written information about neonatal infection before discharge ^19^ | Bloodstream infections | Organisational | Hospital facility | Management |
|  | Evidence of local arrangements for children and young people with suspected bacterial meningitis or meningococcal septicaemia to receive intravenous or intraosseous antibiotics within an hour of arrival at hospital ^19^ | Bloodstream infections | Organisational | Hospital facility | Management |
|  | Infectious disease specialist consultation should be performed in patients with SAB ^32^ | Bloodstream infections | Organisational | Hospital facility | Management |
|  | Heart ultrasound (TEE) within 10 days of collection of first blood culture that became positive (etiologies of bacteremia/sepsis: Staphylococcus aureus, streptococci, enterococci (non-nosocomial), HACEK) ^23^ | Bloodstream infections | Organisational | Hospital facility | Management |
|  | Patients with skin and soft tissue infection undergo documented daily clinical assessment by the OPAT team 7 days per week unless treated with long acting IV agent ^41^ | Skin infections | Clinical | OPAT | Management |
|  | Proportion of patients seeking treatment for acne that should be prescribed oral antibiotics ^7^ | Skin infections | Clinical | Primary health care | Antibiotic prescribing/dispensing |
|  | The number of weeks that antibiotics should be prescribed for in those receiving an antibiotic prescription for their acne ^7^ | Skin infections | Clinical | Primary health care | Antibiotic prescribing/dispensing |
|  | Usage of systemic antibiotics in removal of calculus (dentistry) ^34^ | Dental infections | Clinical | Primary health care | Antibiotic prescribing/dispensing |
|  | Written, locally consented practice guidelines for surgical prophylaxis available and updated (not older than 2 years) available ^23^ | Surgical prophylaxis | Organisational | Hospital facility | Management |
|  | Does your facility audit or review surgical antimicrobial prophylaxis choice and duration? ^26^ | Surgical prophylaxis | Organisational | Hospital facility | Management |
|  | The treatment plan of patients who receive in excess of 1 week of antimicrobial therapy should be regularly reviewed by the OPAT specialist nurse and physician (narrow spectrum antibiotics, intravenous—oral switch) in conjunction/consultation with the referring specialist, as necessary ^61^ | NA | Clinical | OPAT | Antibiotic prescribing/dispensing |
|  | Outpatients and OPAT patients with an antibiotic prescription should be educated on how to take it, on the dosage, on expected side effects, and on the natural history of the disease ^11^ | NA | Clinical | OPAT | Management |
|  | Dose and dosing interval of systemic antibiotic therapy should be adapted to renal function ^24^ | NA | Clinical | General | Antibiotic prescribing/dispensing |
|  | Due to an improvement in mild renal impairment review and adjustment of dosage regimen is documented ^52^ | NA | Clinical | General | Management |
|  | Due to an improvement in moderate renal impairment review and adjustment of dosage regimen is documented ^52^ | NA | Clinical | General | Management |
|  | Due to an improvement in severe renal impairment review and adjustment of dosage regimen is documented ^52^ | NA | Clinical | General | Management |
|  | Blood monitoring with full blood count, renal/liver function, CRP taken weekly as minimum whilst on IV antibiotic or oral linezolid or as per drug monographs for other oral antibiotic regimens ^41^ | NA | Clinical | General | Management |
|  | The measured gentamicin level is documented ^52^ | NA | Clinical | General | Management |
|  | The vancomycin level is taken at the correct time ^52^ | NA | Clinical | General | Management |
|  | The measured vancomycin level is documented ^52^ | NA | Clinical | General | Management |
|  | If antibiotic Therapeutic Drug Monitoring levels are not in the reference range, doses should be adjusted appropriately after the results become available ^39^ | NA | Clinical | General | Antibiotic prescribing/dispensing |
|  | Therapeutic Drug Monitoring levels of antibiotics should be documented in the medical records ^39^ | NA | Clinical | General | Management |
|  | Dose optimization (pharmacokinetics/pharmacodynamics) to optimize the treatment of organisms with reduced susceptibility? ^26^ | NA | Clinical | General | Antibiotic prescribing/dispensing |
|  | Number of deaths during hospitalization / Total number of hospitalizations ^3^ | NA | Clinical | Hospital facility | Outcome |
|  | Number of healthcare-associated infections (MDR organisms (e.g.MRSA, ESBL-E/CPE,MDR Pseudomonas and Acinetobacter spp., vancomycin-resistant enterococci) in a period of time / Total number of patient-days within that period x 100 000 ^3^ | NA | Clinical | Hospital facility | Outcome |
|  | C. difficile hospital-onset rates by reporting acute trust and financial year ^2^ | NA | Clinical | Hospital facility | Outcome |
|  | No. of patients with specific drug-resistant organisms ^27^ | NA | Clinical | General | Outcome |
|  | Mortality related to antimicrobial-resistant organisms ^27^ | NA | Clinical | General | Outcome |
|  | Number of non-duplicated infections by type of MDR in a period x 1000 / Total number of patient days within that period ^9^ | NA | Clinical | General | Outcome |
|  | Patients with a history of anaphylaxis (wheezing, collapse or an itchy rapid onset, urticarial rash) immediately after penicillin therapy should not receive antimicrobial treatment with a beta lactam antimicrobial is prescribed alternative drug class ^52^ | NA | Clinical | General | Antibiotic prescribing/dispensing |
|  | Patients with a history of minor rash (non-confluent, non-pruritic, restricted to a small area of the body, or occurring more than 72 h after penicillin administration) is prescribed a cephalosporin or carbapenem if indicated ^52^ | NA | Clinical | General | Antibiotic prescribing/dispensing |
|  | Allergy status should be taken into account when antibiotics are prescribed ^52^ | NA | Clinical | General | Antibiotic prescribing/dispensing |
|  | When prescribing antibiotics in the ED, the following should be documented in the medical record:  Allergy status (including nature and severity and date the reaction occurred) ^31^ | NA | Clinical | Hospital facility | Management |
|  | Percentage of antibiotic items contained in predefined list (health authority, primary care group, or practice formulary) ^56^ | NA | Clinical | General | Management |
|  | AB consumption feedback to the ward at least 1×/year ^4^ | NA | Clinical | Hospital facility | Management |
|  | Prospective drug use evaluation on the wards by AB officer at least 1 drug/annually ^4^ | NA | Clinical | Hospital facility | Management |
|  | Other resistance rates and corresponding incidence figures (for clinical isolates) available at least once per year for several clinical services (divisions/departments) ^23^ | NA | Clinical | General | Outcome |
|  | Allergy status (including nature and severity) of the patient should be documented in the medical records when antibiotics are prescribed ^39^ | NA | Clinical | General | Management |
|  | There should be a structured OPAT (outpatient parenteral antibiotic therapy) program to provide a framework for safe and effective care ^61^ | NA | Organisational | OPAT | Management |
|  | There should be a formal OPAT care team. The OPAT care team should include an ID specialist or physician knowledgeable about IDs and the use of antimicrobials in OPAT; a nurse who is an expert in intravenous therapy, access devices, and OPAT; and a pharmacist knowledgeable about OPAT ^61^ | NA | Organisational | OPAT | Management |
|  | The OPAT team should have an identifiable, medically qualified lead clinician who has identified time for OPAT in their job plan ^61^ | NA | Organisational | OPAT | Management |
|  | There should be a policy on patient selection criteria for OPAT. The following key aspects of patient selection should be taken into account: patients are willing to comply with the follow-up plan, there is an appropriate home environment/adequate support, there are no clinical contraindications to discharge from the hospital, no intravenous—oral switch is possible, and there is patient and caregiver understanding ^61^ | NA | Organisational | OPAT | Management |
|  | There should be a policy that outlines the responsibilities of OPAT team members ^61^ | NA | Organisational | OPAT | Management |
|  | A competent member of the OPAT team should perform the initial assessment ^61^ | NA | Organisational | OPAT | Management |
|  | An OPAT ID physician consultation should take place prior to intravenous access device placement ^61^ | NA | Organisational | OPAT | Management |
|  | Patients and caregivers should be given the opportunity to decline or accept this mode (OPAT) of therapy ^61^ | NA | Organisational | OPAT | Management |
|  | Patients and their families should be informed about OPAT. The information they get should, at a minimum, at least include benefits, side effects, potential complications, vascular access/sterile techniques, responsible physician until patients seen in clinic, instructions for emergencies, antimicrobial use, patient responsibilities, nature of OPAT, contact lists, and use of antibiotics (eg, storage conditions) ^61^ | NA | Organisational | OPAT | Management |
|  | There should be communication between the OPAT team and other stakeholders. These stakeholders should, at a minimum, at least include a general practitioner, community team (when appropriate), and referring clinician. At a minimum, communication with stakeholders should include notification of acceptance into the OPAT program, notification of completion of therapy, and notification of complications ^61^ | NA | Organisational | OPAT | Management |
|  | The OPAT plan should be documented in the discharge summary ^61^ | NA | Organisational | OPAT | Management |
|  | The program outcome of patients receiving OPAT should be monitored (eg, therapy completed as planned/therapy not completed as planned because of...) ^61^ | NA | Organisational | OPAT | Management |
|  | Antibiotic use of patients receiving OPAT should be monitored (eg, Completed as planned/not Completed as planned because of...) ^61^ | NA | Organisational | OPAT | Management |
|  | The survival status of patients who received OPAT should be documented (eg, patient alive, died of infection, died of other causes, lost to follow-up, or status unknown) ^61^ | NA | Organisational | OPAT | Management |
|  | The satisfaction status/experiences of patients receiving OPAT Should be monitored ^61^ | NA | Organisational | OPAT | Management |
|  | The OPAT treatment plan should include the following items / choice, dose, frequency, duration, and follow-up plan ^61^ | NA | Organisational | OPAT | Management |
|  | The OPAT team should select the drug delivery device in agreement with the home health agency ^61^ | NA | Organisational | OPAT | Management |
|  | The OPAT team should monitor quality indicators for OPAT care and make these data available ^61^ | NA | Organisational | OPAT | Management |
|  | Patient educational material should be available in written or in multimedia form ^61^ | NA | Organisational | OPAT | Management |
|  | There should be an OPAT treatment and monitoring plan. The OPAT treatment and monitoring plan should include, ata minimum, indication, antibiotic name, dose, frequency, duration, type of administration (eg- continuous infusion or bolus infusion), and access device used (ego peripherally inserted central catheter, tunneled catheter) ^61^ | NA | Organisational | OPAT | Management |
|  | OPAT antibiotics should be correctly stored, prepared, reconstituted, dispensed and administered ^61^ | NA | Organisational | OPAT | Management |
|  | The first dose of a new antibiotic in an OPAT should be administered in a supervised setting ^61^ | NA | Organisational | OPAT | Management |
|  | OPAT antibiotics should be regularly reviewed to optimize speed of intra- venous-to-oral switch ^61^ | NA | Organisational | OPAT | Antibiotic prescribing/dispensing |
|  | The OPAT programme should be accredited or certified ^61^ | NA | Organisational | OPAT | Management |
|  | Decision to offer OPAT or not is documented ^41^ | NA | Organisational | OPAT | Management |
|  | Documentation to show that (i) both GP and referrer informed of acceptance onto OPAT within 24 hours of commencing OPAT and that (ii) both referrer and GP informed on completion of OPAT, with follow-up management plan documented ^41^ | NA | Organisational | OPAT | Management |
|  | Documentation that the patient received an OPAT Patient Information Leaflet incorporating treatment received and access to 24 hr emergency care ^41^ | NA | Organisational | OPAT | Management |
|  | Documented OPAT treatment plan that includes BSAC goals and proposed duration of therapy is in place within one week of commencing OPAT ^41^ | NA | Organisational | OPAT | Management |
|  | In non-inpatient settings, intravenous antibiotics should be delivered within a formal OPAT service with clear pathways for early discharge or admission avoidance, in order to ensure patient safety ^57^ | NA | Organisational | OPAT | Management |
|  | The OPAT team should have clear managerial and clinical governance lines of responsibility ^57^ | NA | Organisational | OPAT | Management |
|  | OPAT teams should develop local algorithms for novel treatment strategies, for example, longer acting antimicrobials, new infusion devices, etc. ^57^ | NA | Organisational | OPAT | Management |
|  | OPAT services should consider the role of telemedicine for supporting suitably identified patients at home ^57^ | NA | Organisational | OPAT | Management |
|  | Lead clinical responsibility for patients receiving OPAT should be agreed between the referring clinician and OPAT clinician and documented ^57^ | NA | Organisational | OPAT | Management |
|  | OPAT should be part of a comprehensive infection and antimicrobial stewardship service, in order to maximize opportunities for identification and selection of suitable patients and to optimize appropriate management and minimize unintended consequences of antimicrobial therapy ^57^ | NA | Organisational | OPAT | Management |
|  | It is the responsibility of the infection specialist to agree specific infection-related inclusion and exclusion criteria for OPAT. These should incorporate specific infection severity criteria where appropriate. ^57^ | NA | Organisational | OPAT | Management |
|  | There should be agreed and documented OPAT patient suitability criteria incorporating physical, social and logistic criteria. These should take into account additional risk factors for treatment failure, for example, co-morbidities, lifestyle issues, etc. These should be documented for each patient. ^57^ | NA | Organisational | OPAT | Management |
|  | The treatment plan is the responsibility of the OPAT infection specialist, following discussion with the referring clinician. It should include choice and dose of antimicrobial agent, frequency of administration and duration of therapy and, where appropriate, should take into account flexibility based on clinical response. ^57^ | NA | Organisational | OPAT | Management |
|  | It is the responsibility of the OPAT team to ensure correct and continued prescription of antimicrobials during OPAT, but prescriptions may be written by the referring team under the direction of the OPAT team. Pre-agreed drug choice and dosage for certain conditions (e.g. soft tissue infection in the context of a patient group direction) is acceptable. ^57^ | NA | Organisational | OPAT | Management |
|  | It is the responsibility of the OPAT team to advise on appropriate follow-up for toxicity, compliance and outcome monitoring for those patients recommended by the OPAT team to receive complex oral antibiotic regimens (in place of intravenous therapy). Follow-up of such patients may be best addressed in the immediate post-discharge phase through existing multi-disciplinary OPAT services working within the GPR framework. ^57^ | NA | Organisational | OPAT | Management |
|  | Prescribing for individuals within OPAT should be assessed by an antimicrobial pharmacist ^57^ | NA | Organisational | OPAT | Management |
|  | Insertion and care of the intravascular access device must comply with published RCN standards, and with local and national infection prevention and control guidance ^57^ | NA | Organisational | OPAT | Management |
|  | A member of the OPAT team with the appropriate competencies is responsible for selection of the drug delivery device; use of these must comply with published RCN standards and local hospital guidelines. ^57^ | NA | Organisational | OPAT | Management |
|  | Antimicrobial agents should only be used in pumps or elastomeric devices if there are robust drug stability data meeting the standards of the NHS ‘Standard Protocol for Deriving and Assessment of Stability’ ^57^ | NA | Organisational | OPAT | Antibiotic prescribing/dispensing |
|  | Training of patients or carers in the administration of intravenous medicines must comply with published RCN standards and should be carried out by a member of the OPAT team with the relevant competencies. Both the OPAT nurse specialist and patient/carer must be satisfied of competence and this should be documented. ^57^ | NA | Organisational | OPAT | Management |
|  | All administered doses of intravenous antimicrobial therapy should be documented on a medication card or equivalent, including doses administered out of hospital ^57^ | NA | Organisational | OPAT | Management |
|  | There should be a weekly multidisciplinary meeting/virtual ward round, including as a minimum the OPAT specialist nurse, OPAT physician, medical infection specialist and antimicrobial pharmacist, to discuss progress (including safety monitoring and outcome) of patients receiving OPAT ^57^ | NA | Organisational | OPAT | Management |
|  | Patients receiving in excess of 1 week of antimicrobial therapy should be regularly reviewed by a member of the OPAT team, in addition to discussion at the weekly multi-disciplinary team meeting. The frequency and type of review should be agreed locally. ^57^ | NA | Organisational | OPAT | Antibiotic prescribing/dispensing |
|  | The OPAT team is responsible for monitoring clinical response to antimicrobial management and blood investigations, and for reviewing the treatment plan, in conjunction/consultation with the referring specialist as necessary ^57^ | NA | Organisational | OPAT | Management |
|  | There should be a mechanism in place for urgent discussion and review of emergent clinical problems during therapy according to clinical need. There should be a clear pathway for 24 h immediate access to advice/review/admission for OPAT patients and this should be communicated to the patient both verbally and in writing. ^57^ | NA | Organisational | OPAT | Management |
|  | Data on OPAT patients should be recorded prospectively for service improvement and quality assurance including auditing and benchmarking. A local database would facilitate this process. This information should be shared with all relevant stakeholders, including referring clinicians and general practitioners and may contribute to a national registry. ^57^ | NA | Organisational | OPAT | Management |
|  | Standard outcome criteria should be used on completion of intravenous therapy and these should relate to patient-specific aims of therapy. Data on readmissions, death during OPAT, adverse drug reactions, vascular access complications and healthcare-associated infections, e.g. Clostridioides difficile associated diarrhoea and Staphylococcus aureus bacteraemia, should also be recorded. ^57^ | NA | Organisational | OPAT | Management |
|  | Risk assessment and audit of individual processes (particularly new processes) should be undertaken as part of the local clinical governance programme ^57^ | NA | Organisational | General | Management |
|  | Regular surveys of patient experience should be undertaken in key patient groups (e.g. short-term treatment groups such as those with soft tissue infection and longer-term treatment groups such as those with bone and joint infection) ^57^ | NA | Organisational | General | Management |
|  | There should be an annual review of the service to ensure compliance with national recommendations ^57^ | NA | Organisational | General | Management |
|  | In patients with no prior history of allergy to antimicrobials in the same class, the first dose of a new parenteral antimicrobial may be administered at home under the supervision of healthcare personnel who are qualified and equipped to respond to anaphylactic reactions ^61^ | NA | Organisational | Primary health care | Management |
|  | Evidence of local arrangements to ensure that microbiology laboratories detecting coliform bacteria as a cause of a urinary tract infection report results differentiated by E. coli or non-E. coli organisms ^19^ | NA | Organisational | General | Management |
|  | Vancomycin blood levels should be measured regularly throughout the course of OPAT treatment (strong recommendation, very low-quality evidence). The optimal frequency of measurement is undefined, but the general practice in the setting of stable renal function is once weekly. ^57^ | NA | Organisational | OPAT | Management |
|  | All patients should have ID expert review prior to initiation of OPAT ^57^ | NA | Organisational | OPAT | Management |
|  | There should be a guideline for vascular access systems used, including site care ^61^ | NA | Organisational | General | Management |
|  | Evidence of a written protocol to ensure that people who need a vascular access device have their risk of infection minimised by the completion of specified procedures necessary for the safe insertion and maintenance of the device and its removal as soon as it is no longer needed ^19^ | NA | Organisational | General | Management |
|  | A care plan is documented for the indwelling intravascular device ^41^ | NA | Organisational | General | Management |
|  | Laboratory results should be delivered to physicians within 24 hours after obtaining material for testing ^60^ | NA | Organisational | General | Management |
|  | Transport time of clinical material to the microbiological laboratory during the week possible within 2 h ^4^ | NA | Organisational | General | Management |
|  | Microbiological laboratory. Written directives concerning specimen storage available ^4^ | NA | Organisational | General | Management |
|  | Quality management of the microbiological laboratory certified (according to ISO) ^4^ | NA | Organisational | General | Management |
|  | Quality management of the microbiological laboratory accredited (by government) +B41 ^4^ | NA | Organisational | General | Management |
|  | The microbiological laboratory should report individual selective susceptibly reports (or antibiograms) adapted to local guidelines ^39^ | NA | Organisational | General | Management |
|  | Is there a microbiologist (laboratory)? ^26^ | NA | Organisational | General | Management |
|  | Do you have access to laboratory/imaging services and to timely results to be able to support the diagnosis of the most common infections at your hospital? ^60^ | NA | Organisational | General | Management |
|  | Written in-house preanalytical requirements for microbiologic samples (including rejection criteria) ^23^ | NA | Organisational | General | Management |
|  | Standard antibiotic treatment guidelines should be available in health facilities ^9^ | NA | Organisational | General | Management |
|  | Local clinical practice guidelines/guides for empirical therapy updated biannually ^4^ | NA | Organisational | General | Management |
|  | Clinical audit of prescribers’ compliance with local clinical guidelines/guide performed by AMT/AB officer ^4^ | NA | Organisational | General | Management |
|  | Local clinical practice guidelines/guide for microbiologically documented therapy available ^4^ | NA | Organisational | General | Management |
|  | Local clinical practice guidelines/guide for microbiologically documented therapy updated biannually ^4^ | NA | Organisational | General | Management |
|  | All experts (infectiologist, microbiologist, pharmacist) involved in the guideline/guide development process ^4^ | NA | Organisational | General | Management |
|  | An evaluation whether an update should be considered for the local antibiotic guideline should be done once a year ^39^ | NA | Organisational | General | Management |
|  | The local guidelines should correspond to the national guideline but should be adapted based on local resistance patterns ^39^ | NA | Organisational | General | Management |
|  | Are these treatment recommendations easily accessible to prescribers on all wards (printed “pocket guide” or electronic summaries at workstations)? ^26^ | NA | Organisational | General | Management |
|  | A local antibiotic guideline should be present in the ED of the health-care facility ^31^ | NA | Organisational | Hospital facility | Management |
|  | Written, locally consented practice guidelines (see above) available and updated (not older than 2 years) ^23^ | NA | Organisational | General | Management |
|  | Written recommendation for parenteral-to-oral switch antimicrobial therapy (prerequisites/criteria and drugs) available and updated (not older than 2 years) ^23^ | NA | Organisational | General | Management |
|  | Locally consented guidelines/decision-making aids electronically available (e.g. via physician's computer, PDA or smartphone) ^23^ | NA | Organisational | General | Management |
|  | Key antibiotics should not be out of stock in health facilities that dispense antibiotics ^9^ | NA | Organisational | General | Management |
|  | Antibiotics in stock should not be beyond the expiry date ^9^ | NA | Organisational | General | Management |
|  | Antibiotics should be adequately conserved and handled in health facilities ^9^ | NA | Organisational | General | Management |
|  | Health facilities should have access to the Summary of Product Characteristics of prescribed antibiotics, written in a local language ^9^ | NA | Organisational | General | Management |
|  | Legislation or regulation that requires antimicrobials for human use to be dispensed only with a prescription from an authorized health worker ^1^ | NA | Organisational | General | Management |
|  | Percentage of prescribers in human medicine who are covered by the system for active feedback on the quality and/or quantity of their antibiotic prescribing ^1^ | NA | Organisational | General | Management |
|  | Countries that have reviewed legislation and regulations within the last five years and have a plan to achieve effective regulation of the manufacture, distribution, supply and administration of antimicrobials ^1^ | NA | Organisational | General | Management |
|  | Stock-outs (non-availability) of specified antibiotics at the central warehouse, regional or district medical stores and distributors ^1^ | NA | Organisational | General | Management |
|  | The written communication should be clear, multidisciplinary (e.g. an integrated care pathway) and available and accessible to all relevant members of the clinical team at all times, including out of hours ^56^ | NA | Organisational | General | Management |
|  | Storage, reconstitution and administration of antimicrobials must comply with published Royal Pharmaceutical Society/Royal College of Nursing (RCN) standards and with local hospital clinical pharmacy standards ^57^ | NA | Organisational | General | Management |
|  | Service internal standards for initial treatment with antibiotics ^29^ | NA | Organisational | General | Management |
|  | Computerised antibiotic prescription/order form/system available ^4^ | NA | Organisational | General | Management |
|  | Time-limited drug delivery/automatic stop order available ^4^ | NA | Organisational | General | Management |
|  | AB pocket guidebook or PDA (personal digital assistant) available ^4^ | NA | Organisational | General | Management |
|  | Computer-based clinical decision support for antibiotic prescribing available ^4^ | NA | Organisational | General | Management |
|  | Special request/order form for (selected) antimicrobial drugs available ^4^ | NA | Organisational | General | Management |
|  | Formal mandate for hospital multi-disciplinary antibiotic management team (AMT) existing ^4^ | NA | Organisational | Hospital facility | Management |
|  | Formal mandate for AB officer existing ^4^ | NA | Organisational | General | Management |
|  | Time resources for AMT defined ^4^ | NA | Organisational | General | Management |
|  | Time resources for AB officer defined ^4^ | NA | Organisational | General | Management |
|  | Documented interaction management plan (criteria 12) is followed ^52^ | NA | Organisational | General | Management |
|  | Surveillance of antibiotic use and resistance should be performed at least once per year at the health care facility ^39^ | NA | Organisational | General | Management |
|  | An approval system should be in place for prescriptions of restricted antibiotics at the health care facility ^39^ | NA | Organisational | General | Management |
|  | Clinical outcomes of patients receiving antibiotics should be monitored at the health care facility ^39^ | NA | Organisational | General | Management |
|  | Is it routine practice for specified antimicrobial agents to be approved by a physician or pharmacist in your facility (eg, preauthorization)? ^26^ | NA | Organisational | General | Management |
|  | Has your facility produced a cumulative antimicrobial susceptibility report in the past year? ^26^ | NA | Organisational | General | Management |
|  | Does your facility monitor if the indication is captured in the medical record for all antimicrobial prescriptions? ^26^ | NA | Organisational | General | Management |
|  | Are results of antimicrobial audits or reviews communicated directly with prescribers? ^26^ | NA | Organisational | General | Management |
|  | Are results of audits/reviews of the quality/appropriateness of antimicrobial use communicated directly with prescribers? ^60^ | NA | Organisational | General | Management |
|  | AMS team represented in the drugs and therapeutic committee ^23^ | NA | Organisational | General | Management |
|  | Minimum of 2 AMS team meetings annually (minuted) ^23^ | NA | Organisational | General | Management |
|  | ABS strategic report to D&T and hospital management includes quantitative objectives with selected performance indicators ^23^ | NA | Organisational | General | Management |
|  | Evidence of local arrangements and processes to ensure that all prescribers document the clinical indication, dose and duration of treatment in patients' records when prescribing an antimicrobial ^19^ | NA | Organisational | General | Management |
|  | A documented 24 hour accessible management plan is in place ^41^ | NA | Organisational | General | Management |
|  | Documentation of assessment of antimicrobial prescriptions by an antimicrobial/infection pharmacist ^41^ | NA | Organisational | General | Management |
|  | The clinical response and treatment plan is documented at a weekly multidisciplinary team (MDT)/ virtual ward round ^41^ | NA | Organisational | Hospital facility | Management |
|  | Clinical outcome is recorded as per the BSAC Good Practice Recommendations ^41^ | NA | Organisational | General | Management |
|  | Health facilities should keep adequate records of dispensed key antibiotics ^9^ | NA | Organisational | General | Management |
|  | Does your facility have a written policy that requires prescribers to document an indication in the medical record or during order entry for all antimicrobial prescriptions? ^26^ | NA | Organisational | General | Management |
|  | Is there a document clearly defining the procedures of collaboration of the antimicrobial stewardship team/committee with the infection prevention and control team/committee? ^60^ | NA | Organisational | General | Management |
|  | Infection and/or colonization by multidrug- resistant (MDR) organisms explicitly listed in discharge summary ^23^ | NA | Organisational | General | Management |
|  | The gentamicin level is taken at the correct time recommended by the antimicrobial guidelines ^52^ | NA | Organisational | General | Management |
|  | The gentamicin dosage regimen is managed according to the guideline and a management plan is documented ^52^ | NA | Organisational | General | Management |
|  | The vancomycin dosage regimen is managed according to the guideline and a management plan is documented ^52^ | NA | Organisational | General | Management |
|  | Perform therapeutic drug monitoring in patients treated with vancomycin or aminoglycosides ^53^ | NA | Organisational | General | Management |
|  | Therapeutic Drug Monitoring should be performed for antibiotics with a narrow therapeutic spectrum and an increased risk of toxicity according to guidelines ^39^ | NA | Organisational | General | Management |
|  | Therapeutic drug monitoring should be performed when the treatment duration is more than three days for aminoglycosides and more than five days for vancomycin ^62^ | NA | Organisational | General | Management |
|  | Review and adjustment of dosage regimen as recommended in the guidelines is documented in mild renal impairment (CrCl = 20–50 ml/min) ^52^ | NA | Organisational | General | Management |
|  | Review and adjustment of dosage regimen as recommended in the guidelines is documented in moderate renal impairment (CrCl = 10–20 ml/min) ^52^ | NA | Organisational | General | Management |
|  | Review and adjustment of dosage regimen as recommended in the guidelines is documented in severe renal impairment (CrCl <10 ml/min) ^52^ | NA | Organisational | General | Management |
|  | Dosing adjustments for patients with reduced renal function within 2 days ^23^ | NA | Organisational | General | Antibiotic prescribing/dispensing |
|  | Bedside expert consultant advice regarding antibiotics by microbiologist/infectious disease specialist/antibiotic officer on request available on the same day ^4^ | NA | Organisational | Hospital facility | Management |
|  | Regular ward rounds by members of the AMT (multi-disciplinary antibiotic management team) performed (at least weekly) ^4^ | NA | Organisational | Hospital facility | Management |
|  | AB officer or AMT member is member of the drugs and therapeutics committee ^4^ | NA | Organisational | Hospital facility | Management |
|  | Does your facility have a named senior executive officer with accountability for antimicrobial leadership? ^26^ | NA | Organisational | Hospital facility | Management |
|  | Is clinical infectious disease (ID) consultation available at your facility? ^26^ | NA | Organisational | General | Management |
|  | Is there a physician identified as a leader for stewardship activities at your facility? If YES, Are stewardship duties included in the job description and/or annual review? ^26^ | NA | Organisational | General | Management |
|  | Is there a physician identified as a leader for stewardship activities at your facility? If YES, Is this physician trained in infectious diseases, clinical microbiology, and/or antimicrobial stewardship? ^26^ | NA | Organisational | General | Management |
|  | Is there a pharmacist responsible for working to improve antimicrobial use at your facility? If YES, has this pharmacist had specialized training in infectious disease management or stewardship? Are any of the staff below members involved in stewardship activities at your facility? ^26^ | NA | Organisational | General | Management |
|  | Is there an infection preventionist or hospital epidemiologist? ^26^ | NA | Organisational | Hospital facility | Management |
|  | Is there a document clearly defining roles, procedures of collaboration and responsibilities of the antimicrobial stewardship team members? ^59^ | NA | Organisational | General | Management |
|  | Are clinicians, other than those part of the antimicrobial stewardship team (e.g. from the ICU, Internal Medicine and Surgery) involved in the antimicrobial stewardship committee? ^60^ | NA | Organisational | Hospital facility | Management |
|  | In your hospital are there, or do you have access to, trained and experienced healthcare professionals (medical doctor, pharmacist, nurse …) in infection management (diagnosis, prevention and treatment) and stewardship willing to constitute an antimicrobial stewardship team? ^26^ | NA | Organisational | Hospital facility | Management |
|  | Regular ward rounds of members of ABS team together with the attending physicians (at least 3 clinical services/departments, at least 3 times each in the previous 12 months) ^23^ | NA | Organisational | Hospital facility | Management |
|  | A copy of the essential antibiotics list should be available in health facilities ^11^ | NA | Organisational | General | Management |
|  | An antibiotic formulary should be available and updated continuously at the health care facility ^39^ | NA | Organisational | General | Management |
|  | Does your hospital have an antimicrobial formulary (i.e. a list of antimicrobials that have been approved for use in a hospital, specifying whether the drugs are unrestricted, restricted (approval of an antimicrobial stewardship team member is required) or permitted for specific conditions)? ^60^ | NA | Organisational | Hospital facility | Management |
|  | Evidence of support for, and participation in, joint working initiatives beyond mandatory or contractual requirements, to reduce healthcare-associated infections locally ^19^ | NA | Organisational | Hospital facility | Management |
|  | Evidence of local arrangements for people with a urinary catheter, vascular access device or enteral feeding tube, and their family members or carers (as appropriate), to be educated about the safe management of their device or equipment, including techniques to prevent infection ^19^ | NA | Organisational | General | Management |
|  | Training concept for staff ^28^ | NA | Organisational | General | Management |
|  | Staff participation in training in hygiene and prevention of infections ^28^ | NA | Organisational | General | Management |
|  | AMT (multi-disciplinary antibiotic management team) meetings performed at least bi-monthly ^4^ | NA | Organisational | General | Management |
|  | AB policy and progress report disseminated to medical director by AMT/AB officer ^4^ | NA | Organisational | General | Management |
|  | AB policy and progress report disseminated to infection control committee/hygiene team by AMT/AB officer ^4^ | NA | Organisational | General | Management |
|  | AB policy and progress report disseminated to drugs and therapeutics committee by AMT/AB officer ^4^ | NA | Organisational | General | Management |
|  | AB policy plan with quantitative objectives for performance indicators published annually by AMT/AB officer ^4^ | NA | Organisational | General | Management |
|  | ABS-related formal exchange of experiences (e.g. meeting) of AMT with general practitioners min. 1×/year performed ^4^ | NA | Organisational | Primary health care | Management |
|  | ABS-related formal exchange (e.g. meeting) of experiences of AMT with other hospitals min.  1×/year performed ^4^ | NA | Organisational | Hospital facility | Management |
|  | Clinical audit by AB officer for evaluation of prescribers’ compliance with streamlining drugs on days 2–3 ^4^ | NA | Organisational | General | Management |
|  | Concurrent review by AB officer for evaluation of prescribers’ compliance with streamlining drugs on days 2–3 ^4^ | NA | Organisational | General | Management |
|  | Personnel development Participation of AB officer or members of AMT in AB-related symposia, congresses, seminars at least 1×/year ^4^ | NA | Organisational | General | Management |
|  | Prescriber education by passive methods (bulletin, intranet) performed ^4^ | NA | Organisational | General | Management |
|  | Prescriber education by personalised interactive methods (like daily ward rounds) performed ^4^ | NA | Organisational | General | Management |
|  | Basic diagnostic Working relationship by regular meetings once a week between microbiologists and practitioners ^4^ | NA | Organisational | General | Management |
|  | Biannual face-to-face meetings between ICU and microbiology staff in which local resistance rates are discussed ^53^ | NA | Organisational | General | Management |
|  | Educational sessions about practical guidelines should be organized for medical staff and should have a predetermined attendance target ^38^ | NA | Organisational | General | Management |
|  | Medical staff should be educated regarding cross-allergy with cephalosporins in patients with penicillin allergy ^38^ | NA | Organisational | General | Management |
|  | Does your hospital offer a range of educational resources to support staff training on how to optimize antimicrobial prescribing? ^60^ | NA | Organisational | Hospital facility | Management |
|  | Do the antimicrobial stewardship team members receive regular training in antimicrobial prescribing and stewardship? ^60^ | NA | Organisational | General | Management |
|  | Does your hospital have available and up-to-date recommendations for infection management (diagnosis, prevention and treatment), based on international/national evidence-based guidelines and local susceptibility (when possible), to assist with antimicrobial selection (indication, agent, dose, route, duration) for common clinical conditions? ^60^ | NA | Organisational | Hospital facility | Management |
|  | Educational sessions by the ABS team and/or ABS representatives about locally consented guidelines (tailored to division or at least differentiating conservative vs. surgical specialities) at least every other year ^38^ | NA | Organisational | General | Management |
|  | In-house and/or extramural ABS-relevant continuing medical education about antimicrobial therapy and prophylaxis for at least 10% of medical staff who are not ABS representatives (at least 4 documented CME credits relevant for ABS per year) ^22^ | NA | Organisational | General | Management |
|  | ABS-relevant continuing medical education for ABS team members and ABS representatives from clinical services (at least 8 documented CME credits relevant for ABS per year) ^22^ | NA | Organisational | General | Management |
|  | Percentage of acute health care facilities with an antimicrobial stewardship programme in place ^1^ | NA | Organisational | General | Management |
|  | Evidence of local arrangements to deliver an antimicrobial stewardship programme ^18^ | NA | Organisational | General | Management |
|  | Evidence of local arrangements and processes to ensure that individuals and teams responsible for antimicrobial stewardship monitor data and provide feedback on prescribing practice at prescriber, team, organisation and commissioner level ^19^ | NA | Organisational | General | Management |
|  | Audits of antibiotic use by the antibiotic stewardship team should be performed regularly at the health care facility ^38^ | NA | Organisational | General | Management |
|  | A multidisciplinary antibiotic stewardship team appointed by the health care facility management should have meetings at least twice a year and make a report with objectives and selected performance indicators ^38^ | NA | Organisational | General | Management |
|  | Does your facility have a formal organizational structure responsible for antimicrobial stewardship (eg, a multidisciplinary committee focused on appropriate antimicrobial use, pharmacy committee, patient safety committee, or other relevant structure)? ^25^ | NA | Organisational | General | Management |
|  | Has your hospital management formally identified antimicrobial stewardship as a priority objective for the institution and included it in its key performance indicators? ^60^ | NA | Organisational | Hospital facility | Management |
|  | Is there dedicated, sustainable and sufficient budgeted financial support for antimicrobial stewardship activities (e.g., support for salary, training, or IT (information technology) support)? ^25^ | NA | Organisational | General | Management |
|  | Does your hospital follow any (national or international) staffing standards for antimicrobial stewardship activities (e.g. number of full-time equivalent (FTE) per 100 beds for the different members of the antimicrobial stewardship team)? ^25^ | NA | Organisational | Hospital facility | Management |
|  | Does the antimicrobial stewardship committee produce regularly (indicate minimum time) a dedicated report which includes e.g. antimicrobial use data and/or prescription improvement initiatives, with time-committed short term and long-term measurable goals/ targets for optimising antimicrobial use? ^60^ | NA | Organisational | General | Management |
|  | Does your stewardship programme monitor compliance with one or more of the specific interventions put in place by the stewardship team (e.g. indication captured in the medical record for all antimicrobial prescriptions)? ^60^ | NA | Organisational | Hospital facility | Management |
|  | Does your stewardship programme share hospital-specific reports on the quantity of antimicrobials prescribed/dispensed/purchased with prescribers? ^60^ | NA | Organisational | Hospital facility | Management |
|  | Does your stewardship programme share facility-specific reports on antibiotic susceptibility rates with prescribers? ^60^ | NA | Organisational | General | Management |
|  | An antibiotic stewardship programme that comprises measuring and improving antibiotic use should also cover the ED ^30^ | NA | Organisational | Hospital facility | Management |
|  | Antibiotic resistance data regarding MRSA analysed and written report provided at least 1×/year ^4^ | NA | Organisational | General | Management |
|  | Antibiotic resistance data regarding ESBL analysed at least 1×/year ^4^ | NA | Organisational | General | Management |
|  | Antibiotic resistance data (other than MRSA and ESBL) analysed at least 1×/year ^4^ | NA | Organisational | General | Management |
|  | Are there regular infection and antimicrobial prescribing focused ward rounds in specific departments in your hospital? ^60^ | NA | Organisational | Hospital facility | Management |
|  | Does your hospital monitor the quality of antimicrobial use at the unit and/or hospital wide level? ^60^ | NA | Organisational | Hospital facility | Management |
|  | Does your hospital monitor antibiotic susceptibility rates for a range of key bacteria? ^60^ | NA | Organisational | Hospital facility | Management |
|  | Length of hospital stay ^19^ | NA | Organisational | Hospital facility | Management |
|  | Countries that conduct regular and risk-based post-market surveillance on antimicrobials a / For humans b / For animals (terrestrial, aquatic) ^1^ | NA | Organisational | General | Management |
|  | E. coli bacteraemia hospital-onset counts and rates by NHS acute trust and financial year ^2^ | NA | Organisational | Hospital facility | Management |
|  | Antibiotic Guardians per 100,000 population per calendar year by CCGs ^2^ | NA | Organisational | General | Management |
|  | Antibiotic Guardians per 100,000 population per quarter by CCG ^2^ | NA | Organisational | General | Management |
|  | Evidence of local arrangements to ensure that people with a self‑limiting condition, as assessed by a primary care prescriber, receive advice about self-management and adverse consequences of overusing antimicrobials ^19^ | NA | Organisational | General | Management |
|  | Evidence of local arrangements to promote self-management of self‑limiting conditions and raise awareness of risks associated with overusing antimicrobials ^19^ | NA | Organisational | General | Management |
|  | Proportion of presentations in primary care assessed as a self‑limiting condition with a record stating that advice about self‑management was given ^19^ | NA | Organisational | Primary health care | Management |
|  | Proportion of presentations in primary care assessed as a self‑limiting condition with a record stating that advice about the adverse consequences of overusing antimicrobials was given ^19^ | NA | Organisational | Primary health care | Management |
|  | Evidence of local arrangements and processes to ensure that people in hospital who are prescribed an antimicrobial have a microbiological sample taken and their treatment reviewed when the results are available ^19^ | NA | Organisational | Hospital facility | Management |
|  | Proportion of prescribers who receive feedback on their antimicrobial prescribing practice ^19^ | NA | Organisational | General | Management |
|  | Proportion of teams within an organisation that receive feedback on their antimicrobial prescribing practice ^19^ | NA | Organisational | General | Management |
|  | Proportion of organisations within a specified commissioning area that receive feedback on their antimicrobial prescribing practice ^19^ | NA | Organisational | General | Management |
|  | Evidence of local arrangements to ensure that prescribers of antimicrobials in secondary care and dental care settings have access to electronic prescribing systems that link indication with the antimicrobial prescription ^19^ | NA | Organisational | Primary health care | Management |
|  | Proportion of secondary care services using electronic prescribing systems that link the indication with the antimicrobial prescription ^19^ | NA | Organisational | Primary health care | Management |
|  | Proportion of dental practices using electronic prescribing systems that link the indication with the antimicrobial prescription ^19^ | NA | Organisational | Primary health care | Management |
|  | Evidence of local arrangements for hospitals to monitor healthcare‑associated infections and other infections of local relevance ^19^ | NA | Organisational | Hospital facility | Management |
|  | Evidence of local arrangements for the results of monitoring healthcare‑associated infections and other infections of local relevance to be used across the organisation to inform and review objectives for quality improvement ^19^ | NA | Organisational | Hospital facility | Management |
|  | Evidence of local arrangements for hospitals to monitor the risk of healthcare‑associated infections from incidents and outbreaks in the community ^19^ | NA | Organisational | General | Management |
|  | Evidence of local arrangements for collaborative working between hospitals and other local health and social care organisations to investigate and manage the risks of healthcare‑associated infection from incidents and outbreaks in the community ^19^ | NA | Organisational | General | Management |
|  | Evidence of local arrangements to ensure all staff have clear objectives in relation to infection prevention and control that are linked to board‑level objectives ^19^ | NA | Organisational | General | Management |
|  | Evidence of local arrangements to ensure all staff have an appraisal and development plan that cover infection prevention and control ^19^ | NA | Organisational | General | Management |
|  | Proportion of hospital staff who have individual infection prevention and control objectives that are linked to board‑level objectives ^19^ | NA | Organisational | Hospital facility | Management |
|  | Proportion of hospital staff who have an appraisal of their infection prevention and control objectives ^19^ | NA | Organisational | Hospital facility | Management |
|  | Proportion of hospital staff who have a development plan that includes infection prevention and control ^19^ | NA | Organisational | Hospital facility | Management |
|  | Evidence of local arrangements for involving infection prevention and control teams in the building and refurbishment of facilities in the hospital. Examples of evidence may include protocols covering infection prevention and control in the built environment; estate department procedures to engage infection prevention and control teams in new build and refurbishment projects; building and refurbishment project plans and schedules of work that show the involvement of infection prevention and control teams; and records of completed building and refurbishment works that show whether infection prevention and control requirements have been met ^19^ | NA | Organisational | General | Management |
|  | Evidence of local arrangements for involving infection prevention and control teams in the maintenance of facilities in the hospital. Examples of evidence may include protocols covering infection prevention and control in the built environment; estate department procedures to engage infection prevention and control teams in maintenance works; maintenance plans and schedules that show the involvement of infection prevention and control teams; and records of completed maintenance works that show whether infection prevention and control requirements have been met. ^19^ | NA | Organisational | General | Management |
|  | Evidence of local arrangements to ensure information about any infections and associated treatments for people admitted to, discharged from, or transferred between or within hospitals, is shared with the health and social care staff responsible for the ongoing care ^19^ | NA | Organisational | General | Management |
|  | Proportion of admissions to hospital, including transfers of patients from other hospitals, where information on infections and associated treatments is received ^19^ | NA | Organisational | Hospital facility | Management |
|  | Proportion of discharges from hospital, including transfers of patients to other hospitals, where information on infections and associated treatments is provided to health and social care staff responsible for ongoing care ^19^ | NA | Organisational | Hospital facility | Management |
|  | Proportion of transfers of patients within a hospital where information on infections and associated treatments is provided to health care staff responsible for ongoing care ^19^ | NA | Organisational | Hospital facility | Management |
|  | Evidence of local antibiotic formularies governing the use of antibiotics to ensure that people are prescribed antibiotics appropriately ^19^ | NA | Organisational | General | Management |
|  | Evidence that the organisation's board is up to date with, and has a working knowledge and understanding of, infection prevention and control ^19^ | NA | Organisational | General | Management |
|  | Patient satisfaction with information received about how to recognise re-infection and to seek medical advice straight away ^19^ | NA | Organisational | General | Management |
|  | The first dose of all new IV antimicrobial therapy is administered and documented by an appropriately trained healthcare professional with facilities for anaphylaxis resuscitation ^41^ | NA | Organisational | General | Management |
|  | Is there a formal procedure for a physician, pharmacist, or other staff member to review the appropriateness of an antimicrobial at or after 48 hours from the initial order (post-prescription review)? ^26^ | NA | Organisational | General | Management |
|  | Standardized criteria for changing from intravenous to oral antimicrobial therapy in appropriate situations? ^26^ | NA | Organisational | General | Management |
|  | Patients in the ED who are discharged with an antibiotic prescription should be educated on / - How to take it - The dosage - The expected side effects and potential interaction with other therapies - Duration of treatment ^31^ | NA | Organisational | Hospital facility | Management |
|  | % key antimicrobials available ^9^ | NA | Organisational | General | Management |
|  | The OPAT ID physician should specify infection-related inclusion and exclusion criteria for OPAT ^61^ | NA | Workforce | OPAT | Management |
|  | Patients or carers (self)-administering IV medicines have competencies signed off by an OPAT nurse specialist ^41^ | NA | Workforce | OPAT | Management |
|  | Initial assessment for OPAT should be performed by a competent member of the OPAT team ^61^ | NA | Workforce | OPAT | Management |
|  | Each member of the OPAT team is responsible for personal continuing professional development relating to best clinical practice ^57^ | NA | Workforce | OPAT | Management |

ABS = American Board of Surgery; AMT = antibiotic management team; CCGs = Clinical Commissioning Group; ED = emergency department; ESBL = Extended-spectrum beta-lactamase; ICU= intensive care unit; MRSA = Methicillin-resistant Staphylococcus aureus; NA = not applicable; OPAT = Outpatient parenteral antibiotic therapy; SAB = *Staphylococcus aureus* bacteremia.

1. World Health Organization, Food and Agriculture Organization of the United Nations, World Organisation for Animal Health. Monitoring and evaluation of the global action plan on antimicrobial resistance: framework and recommended indicators. Published online 2019. https://iris.who.int/bitstream/handle/10665/325006/9789241515665-eng.pdf?sequence=1
2. UKHSA. AMR local indicators. Published online 2023. https://fingertips.phe.org.uk/profile/amr-local-indicators
3. World Health Organization. Antimicrobial stewardship programmes in health-care facilities in low- and middle-income countries: a practical toolkit. Published online 2019. https://apps.who.int/iris/bitstream/handle/10665/329404/9789241515481-eng.pdf?sequence=1&isAllowed=y
4. on behalf of members of the Antibiotic Strategy International (ABS) Quality Indicators Team, Buyle FM, Metz-Gercek S, et al. Development and validation of potential structure indicators for evaluating antimicrobial stewardship programmes in European hospitals. Eur J Clin Microbiol Infect Dis. 2013;32(9):1161-1170. doi:10.1007/s10096-013-1862-4
5. Centers for Medicare & Medicaid Services. Quality Measures Related to Management of Adult ID. Published online 2018. https://qpp.cms.gov/mips/explore-measures?tab=qualityMeasures&py=2018
6. Saust LT, Bjerrum L, Arpi M, Hansen MP. Quality indicators for the diagnosis and antibiotic treatment of acute respiratory tract infections in general practice: a RAND Appropriateness Method. Scandinavian Journal of Primary Health Care. 2017;35(2):192-200. doi:10.1080/02813432.2017.1333305
7. Smith DRM, Dolk FCK, Pouwels KB, Christie M, Robotham JV, Smieszek T. Defining the appropriateness and inappropriateness of antibiotic prescribing in primary care. Journal of Antimicrobial Chemotherapy. 2018;73(suppl_2):ii11-ii18. doi:10.1093/jac/dkx503
8. Schouten JA, Hulscher MEJL, Wollersheim H, et al. Quality of Antibiotic Use for Lower Respiratory Tract Infections at Hospitals: (How) Can We Measure It? *Clinical Infectious Diseases*. 2005;41(4):450-460. doi:10.1086/431983
9. Pan American Health Organization. Recommendations for Implementing Antimicrobial Stewardship Programs in Latin America and the Caribbean: Manual for Public Health Decision-Makers. https://iris.paho.org/handle/10665.2/49645
10. Adriaenssens N, Coenen S, Tonkin-Crine S, et al. European Surveillance of Antimicrobial Consumption (ESAC): disease-specific quality indicators for outpatient antibiotic prescribing. BMJ Quality & Safety. 2011;20(9):764-772. doi:10.1136/bmjqs.2010.049049
11. Le Maréchal M, Tebano G, Monnier AA, et al. Quality indicators assessing antibiotic use in the outpatient setting: a systematic review followed by an international multidisciplinary consensus procedure. Journal of Antimicrobial Chemotherapy. 2018;73(suppl_6):vi40-vi49. doi:10.1093/jac/dky117
12. NCQA. HEDIS (Healthcare Effectiveness Data and Information Set): HEDIS Measures and Technical Resources. https://www.ncqa.org/hedis/measures/antibiotic-utilization/
13. Cottrell J, Yip J, Campisi P, et al. Quality indicators for the diagnosis and management of pediatric tonsillitis. International Journal of Pediatric Otorhinolaryngology. 2020;139:110441. doi:10.1016/j.ijporl.2020.110441
14. Hansen MP, Bjerrum L, Gahrn-Hansen B, Jarbol DE. Quality indicators for diagnosis and treatment of respiratory tract infections in general practice: A modified Delphi study. Scandinavian Journal of Primary Health Care. 2010;28(1):4-11. doi:10.3109/02813431003602724
15. Van Roosmalen MS, Braspenning JCC, De Smet PAGM, Grol RPTM. Antibiotic prescribing in primary care: first choice and restrictive prescribing are two different traits. Quality and Safety in Health Care. 2007;16(2):105-109. doi:10.1136/qshc.2006.018580
16. Cottrell J, Yip J, Chan Y, et al. Quality Indicators for the Diagnosis and Management of Acute Bacterial Rhinosinusitis. Am J Rhinol Allergy. 2020;34(4):519-531. doi:10.1177/1945892420912158
17. Giesen P, Willekens M, Mokkink H, Braspenning J, Van Den Bosch W, Grol R. Out-of-hours primary care: development of indicators for prescribing and referring. International Journal for Quality in Health Care. 2007;19(5):289-295. doi:10.1093/intqhc/mzm027
18. NHS England. Commissioning for Quality and Innovation (CQUIN): 2023/24 guidance. Published online 2023. https://www.england.nhs.uk/nhs-standard-contract/cquin/cquin-23-24/
19. NICE. Guidance, NICE advice and quality standards.
20. Agency for Healthcare Research and Quality. Quality Improvement and monitoring. https://qualityindicators.ahrq.gov/
21. Farida H, Rondags A, Gasem MH, et al. Development of quality indicators to evaluate antibiotic treatment of patients with community‐acquired pneumonia in I ndonesia. Tropical Med Int Health. 2015;20(4):501-509. doi:10.1111/tmi.12452
22. Li W, Zeng L, Li J, et al. Development of indicators for assessing rational drug use to treat community-acquired pneumonia in children in hospitals and clinics: A modified Delphi study. Medicine. 2017;96(51):e9308. doi:10.1097/MD.0000000000009308
23. Thern J, De With K, Strauss R, Steib-Bauert M, Weber N, Kern WV. Selection of hospital antimicrobial prescribing quality indicators: a consensus among German antibiotic stewardship (ABS) networkers. Infection. 2014;42(2):351-362. doi:10.1007/s15010-013-0559-z
24. Hermanides HS, Hulscher MEJL, Schouten JA, Prins JM, Geerlings SE. Development of Quality Indicators for the Antibiotic Treatment of Complicated Urinary Tract Infections: A First Step to Measure and Improve Care. Clinical Infectious Diseases. 2008;46(5):703-711. doi:10.1086/527384
25. Korom RR, Onguka S, Halestrap P, McAlhaney M, Adam M. Brief educational interventions to improve performance on novel quality metrics in ambulatory settings in Kenya: A multi-site pre-post effectiveness trial. Sands JM, ed. *PLoS ONE*. 2017;12(4):e0174566. doi:10.1371/journal.pone.0174566
26. Pollack LA, Plachouras D, Sinkowitz-Cochran R, et al. A Concise Set of Structure and Process Indicators to Assess and Compare Antimicrobial Stewardship Programs Among EU and US Hospitals: Results From a Multinational Expert Panel. *Infect Control Hosp Epidemiol*. 2016;37(10):1201-1211. doi:10.1017/ice.2016.115
27. Morris AM, Brener S, Dresser L, et al. Use of a Structured Panel Process to Define Quality Metrics for Antimicrobial Stewardship Programs. Infect Control Hosp Epidemiol. 2012;(5):500-506. doi:10.1086/665324
28. Harvey EJ, Hand K, Weston D, Ashiru-Oredope D. Development of National Antimicrobial Intravenous-to-Oral Switch Criteria and Decision Aid. *JCM*. 2023;12(6):2086. doi:10.3390/jcm12062086
29. Bramesfeld A, Wrede S, Richter K, et al. Development of quality indicators and data assessment strategies for the prevention of central venous catheter-related bloodstream infections (CRBSI). *BMC Infect Dis*. 2015;15(1):435. doi:10.1186/s12879-015-1200-9
30. Berenholtz SM, Pronovost PJ, Ngo K, et al. Developing Quality Measures for Sepsis Care in the ICU. *The Joint Commission Journal on Quality and Patient Safety*. 2007;33(9):559-568. doi:10.1016/S1553-7250(07)33060-2
31. Schoffelen T, Schouten J, Hoogerwerf J, et al. Quality indicators for appropriate antimicrobial therapy in the emergency department: a pragmatic Delphi procedure. *Clinical Microbiology and Infection*. 2021;27(2):210-214. doi:10.1016/j.cmi.2020.10.027
32. Ten Oever J, Jansen JL, Van Der Vaart TW, Schouten JA, Hulscher MEJL, Verbon A. Development of quality indicators for the management of Staphylococcus aureus bacteraemia. *Journal of Antimicrobial Chemotherapy*. 2019;74(11):3344-3351. doi:10.1093/jac/dkz342
33. Vera P, Palomar M, Álvarez-Lerma F. Quality indicators on the use of antimicrobials in critically ill patients. Medicina Intensiva. 2014;38(9):567-574. doi:10.1016/j.medin.2014.04.009
34. Hussein RJ, Krohn R, Kaufmann-Kolle P, Willms G. Quality indicators for the use of systemic antibiotics in dentistry. *Zeitschrift für Evidenz, Fortbildung und Qualität im Gesundheitswesen*. 2017;122:1-8. doi:10.1016/j.zefq.2017.04.007
35. Australian Commission on Safety and Quality in Health Care: Indicators for the Antimicrobial Stewardship Clinical Care Standard. https://www.safetyandquality.gov.au/our-work/clinical-care-standards/antimicrobial-stewardship-clinical-care-standard/indicators#list-of-indicators
36. Afriyie DK, Sefah IA, Sneddon J, et al. Antimicrobial point prevalence surveys in two Ghanaian hospitals: opportunities for antimicrobial stewardship. *JAC-Antimicrobial Resistance*. 2020;2(1):dlaa001. doi:10.1093/jacamr/dlaa001
37. Skosana P, Schellack N, Godman B, et al. A point prevalence survey of antimicrobial utilisation patterns and quality indices amongst hospitals in South Africa; findings and implications. *Expert Review of Anti-infective Therapy*. 2021;19(10):1353-1366. doi:10.1080/14787210.2021.1898946
38. Korea Study Group for Antimicrobial Stewardship (KOSGAP), Kim B, Lee MJ, et al. Development of key quality indicators for appropriate antibiotic use in the Republic of Korea: results of a modified Delphi survey. *Antimicrob Resist Infect Control*. 2021;10(1):48. doi:10.1186/s13756-021-00913-y
39. Monnier AA, Schouten J, Le Maréchal M, et al. Quality indicators for responsible antibiotic use in the inpatient setting: a systematic review followed by an international multidisciplinary consensus procedure. *Journal of Antimicrobial Chemotherapy*. 2018;73(suppl_6):vi30-vi39. doi:10.1093/jac/dky116
40. Coenen S, Ferech M, Haaijer-Ruskamp FM, et al. European Surveillance of Antimicrobial Consumption (ESAC): quality indicators for outpatient antibiotic use in Europe. Quality and Safety in Health Care. 2007;16(6):440-445. doi:10.1136/qshc.2006.021121
41. Scottish Antimicrobial Stewardship Group. Key performance indicators (KPIs) for the management of patients in an outpatient parenteral antimicrobial therapy (OPAT) setting. Published online 2022. https://www.sapg.scot/media/6994/sapg-opat-kpis-final.pdf. https://www.isdscotland.org/Health-Topics/Prescribing-and-Medicines/Publications/2014-10-14/2014-10-14-SAPG-Primary-Care-PI-2013-14-Report.pdf
42. Israel Ministry of Health, Quality and Safety Division, Health Services Research Department. Israeli National Program for Quality Indicators in Community Healthcare (Surgical Site Infections). https://en.israelhealthindicators.org/
43. Organisation for Economic Co-operation and Development. Health Care Quality Indicators - Primary Care. https://www.oecd.org/els/health-systems/hcqi-primary-care.htm
44. Versporten A, Gyssens IC, Pulcini C, et al. Metrics to assess the quantity of antibiotic use in the outpatient setting: a systematic review followed by an international multidisciplinary consensus procedure. Journal of Antimicrobial Chemotherapy. 2018;73(suppl_6):vi59-vi66. doi:10.1093/jac/dky119
45. Stanić Benić M, Milanič R, Monnier AA, et al. Metrics for quantifying antibiotic use in the hospital setting: results from a systematic review and international multidisciplinary consensus procedure. Journal of Antimicrobial Chemotherapy. 2018;73(suppl_6):vi50-vi58. doi:10.1093/jac/dky118
46. Okoth C, Opanga S, Okalebo F, Oluka M, Baker Kurdi A, Godman B. Point prevalence survey of antibiotic use and resistance at a referral hospital in Kenya: findings and implications. Hospital Practice. 2018;46(3):128-136. doi:10.1080/21548331.2018.1464872
47. European Centre for Disease Prevention and Control (ECDC), European Food Safety Authority (EFSA), European Medicines Agency (EMA). Antimicrobial consumption and resistance in bacteria from humans and food‐producing animals. *EFS2*. 2024;22(2). doi:10.2903/j.efsa.2024.8589
48. Pulcini C, Lions C, Ventelou B, Verger P. Drug-specific quality indicators assessing outpatient antibiotic use among French general practitioners. The European Journal of Public Health. 2013;23(2):262-264. doi:10.1093/eurpub/cks100
49. Fernández Urrusuno R, Pedregal González M, Torrecilla Rojas MaA. Antibiotic prescribing patterns and hospital admissions with respiratory and urinary tract infections. Eur J Clin Pharmacol. 2008;64(10):1005-1011. doi:10.1007/s00228-008-0514-6
50. De Bie S, Kaguelidou F, Verhamme KMC, et al. Using Prescription Patterns in Primary Care to Derive New Quality Indicators for Childhood Community Antibiotic Prescribing. *Pediatric Infectious Disease Journal*. 2016;35(12):1317-1323. doi:10.1097/INF.0000000000001324
51. World Health Organization I. How to investigate drug use in health facilities - Selected drug use indicators. Published online 1993
52. Coll A, Kinnear M, Kinnear A. Design of antimicrobial stewardship care bundles on the high dependency unit. *Int J Clin Pharm*. 2012;34(6):845-854. doi:10.1007/s11096-012-9680-9
53. Kallen MC, Roos-Blom MJ, Dongelmans DA, et al. Development of actionable quality indicators and an action implementation toolbox for appropriate antibiotic use at intensive care units: A modified-RAND Delphi study. Pittet V, ed. *PLoS ONE*. 2018;13(11):e0207991. doi:10.1371/journal.pone.0207991
54. Pulcini C, Defres S, Aggarwal I, Nathwani D, Davey P. Design of a “day 3 bundle” to improve the reassessment of inpatient empirical antibiotic prescriptions. *Journal of Antimicrobial Chemotherapy*. 2008;61(6):1384-1388. doi:10.1093/jac/dkn113
55. Oduyebo O, Olayinka A, Iregbu K, et al. A point prevalence survey of antimicrobial prescribing in four Nigerian Tertiary Hospitals. *Ann Trop Pathol*. 2017;8(1):42. doi:10.4103/atp.atp_38_17
56. Campbell SM. Prescribing indicators for UK general practice: Delphi consultation study. *BMJ*. 2000;321(7258):425-428. doi:10.1136/bmj.321.7258.425
57. Chapman ALN, Patel S, Horner C, et al. Updated good practice recommendations for outpatient parenteral antimicrobial therapy (OPAT) in adults and children in the UK. *JAC-Antimicrobial Resistance*. 2019;1(2):dlz026. doi:10.1093/jacamr/dlz026
58. Bateman DN, Eccles M, Campbell M, Soutter J, Roberts SJ, Smith JM. Setting standards of prescribing performance in primary care: use of a consensus group of general practitioners and application of standards to practices in the north of England. *Br J Gen Pract*. 1996;46(402):20-25.
59. Science M, Timberlake K, Morris A, Read S, Le Saux N, on behalf of the Groupe Antibiothérapie en Pédiatrie Canada Alliance for Stewardship of Antimicrobials in Pediatrics (GAP Can ASAP). Quality Metrics for Antimicrobial Stewardship Programs. *Pediatrics*. 2019;143(4):e20182372. doi:10.1542/peds.2018-2372
60. Pulcini C, Binda F, Lamkang AS, et al. Developing core elements and checklist items for global hospital antimicrobial stewardship programmes: a consensus approach. *Clinical Microbiology and Infection*. 2019;25(1):20-25. doi:10.1016/j.cmi.2018.03.033
61. Berrevoets MAH, Ten Oever J, Oerlemans AJM, Kullberg BJ, Hulscher ME, Schouten JA. Quality Indicators for Appropriate Outpatient Parenteral Antimicrobial Therapy in Adults: A Systematic Review and RAND-modified Delphi Procedure. *Clinical Infectious Diseases*. 2020;70(6):1075-1082. doi:10.1093/cid/ciz362
62. Van Den Bosch CMA, Geerlings SE, Natsch S, Prins JM, Hulscher MEJL. Quality Indicators to Measure Appropriate Antibiotic Use in Hospitalized Adults. *Clinical Infectious Diseases*. 2015;60(2):281-291. doi:10.1093/cid/ciu747
